# Ecosystem approach to fisheries: Exploring environmental and trophic effects on Maximum Sustainable Yield (MSY) reference point estimates

**DOI:** 10.1371/journal.pone.0185575

**Published:** 2017-09-28

**Authors:** Rajeev Kumar, Tony J. Pitcher, Divya A. Varkey

**Affiliations:** Institute for the Oceans and Fisheries (formerly Fisheries Center), University of British Columbia, Vancouver, British Columbia, Canada; Sveriges lantbruksuniversitet, SWEDEN

## Abstract

We present a comprehensive analysis of estimation of fisheries Maximum Sustainable Yield (MSY) reference points using an ecosystem model built for Mille Lacs Lake, the second largest lake within Minnesota, USA. Data from single-species modelling output, extensive annual sampling for species abundances, annual catch-survey, stomach-content analysis for predatory-prey interactions, and expert opinions were brought together within the framework of an Ecopath with Ecosim (EwE) ecosystem model. An increase in the lake water temperature was observed in the last few decades; therefore, we also incorporated a temperature forcing function in the EwE model to capture the influences of changing temperature on the species composition and food web. The EwE model was fitted to abundance and catch time-series for the period 1985 to 2006. Using the ecosystem model, we estimated reference points for most of the fished species in the lake at single-species as well as ecosystem levels with and without considering the influence of temperature change; therefore, our analysis investigated the trophic and temperature effects on the reference points. The paper concludes that reference points such as MSY are not stationary, but change when (1) environmental conditions alter species productivity and (2) fishing on predators alters the compensatory response of their prey. Thus, it is necessary for the management to re-estimate or re-evaluate the reference points when changes in environmental conditions and/or major shifts in species abundance or community structure are observed.

## Introduction

Management based on single-species models could tend to ignore the broader context in terms of species interactions and ecological and environmental processes within which the fishery operates [1, 2]. Single-species management is not immune to risks such as by-catch of endangered or protected species [3]. While reviewing multi-species models, Hollowed et al. [4] stressed the need for multi-species models to assess the impact of energy flows in an ecosystem; they argued that competition, predation, and environmental effects were the three fundamental factors that determine the ecosystem dynamics; and therefore, these factors should form a “framework” for multispecies modelling. Moreover, uncertainties associated with the model’s predictions increase to a greater extent when changes in environmental condition perturb the dynamics between key species and modelling exercises that take into account the multitude of interactions among biotic, abiotic, and anthropogenic drivers of an ecosystem [1].

A comparison of management reference points derived from single-species versus ecosystem approaches will allow a systematic comparison of management advice generated from adopting one or the other approach for management. Over the last half-century, MSY, the reference point we estimated, evolved from a target to a limit reference point and continues to be a popular metric [5]. There have been several criticisms of the use of MSY suitability as a management target [6, 7], especially the challenges of estimation with respect to changes in recruitment and multispecies influences in the ecosystem. Mace [8] suggested using an integration of both single-species and multispecies approaches. Adopting MSY as the target resulted in managers treating the metric as the ‘maximum average catch’ and allowing the actual fisheries catch to sometimes overshoot resulting in overfishing [8]. The value of MSY was eventually “reborn” with a new face as a limit reference point [8].

Few works have actually compared single-species (SS) and ecosystem (ES) based estimates of the MSY metric. After a comparison of SSmsy and ESmsy estimates, Walters et al. [9] using an EwE based analysis concluded that focus on single-species methods alone could be detrimental to fisheries, especially for species at higher trophic level. A comparison of reference points generated in a virtual population analysis (VPA) and multi-species VPA (MSVPA), Gislason [10] showed that multispecies calculations provided highly valuable information, though management based on ecosystem reference points was complicated because the value generated by the predator (cod) and its prey species (herring and sprat) were different. Further, expected ecosystem outcomes become more uncertain when environmental changes and fishing pressure occur concurrently. From an SS perspective, Wayte [11] showed that not adjusting reference points in the wake of environmental changes could lead to erroneous management recommendations. Within an ES perspective, Large et al. [12] showed that estimates of ecosystem indicators changed in response to environmental fluctuations and provided an improved understanding of changing ecosystem status.

Temperature is one of the most significant abiotic components of ecosystems because it plays dominant roles in determining the rate of the physiological process of organisms, especially cold-water species in the northern hemisphere [13], and therefore affects the functioning of the food-web. In the present study area (Mille Lacs Lake, Minnesota), Species such as cisco (Coregonus artedi) and burbot (Lota lota) are cold-water stenotherm and therefore are highly sensitive to the changes in the lake water temperature. Moreover, species like smallmouth bass (Micropterus dolomieu), especially young immature bass have higher growth and survival in warmer waters [14]. Adding the effects of temperature while accounting for trophic interactions would help to make the MSY estimates more realistic.

Our four objectives with the analysis were: (1) to estimate the biological reference points, MSY and Fmsy, for the principal fished species present in the study area under two frameworks—(a) Ecosystem MSY (ESmsy), where food-web dynamics were accounted for while estimating the MSY of the target species and (b) traditional single-species MSY (SSmsy), where it was assumed that the population dynamics of non-target species is unaffected as a result of fishing on the target species; (2) to explore the reasons for different values, if any, obtained for SSmsy and ESmsy i.e., the compensation that resulted in higher estimates of ESmsy or the costs, for example competition, that resulted in higher SSmsy levels; (3) to explore the influence of temperature fluctuations on the SSmsy and ESmsy estimates when the temperature forcing-function was applied to the dynamics of three species, cisco, burbot and smallmouth bass, and (4) to conduct a sensitivity analysis on the MSY estimates.

## Materials and methods

### Study site

**Fig 1:**
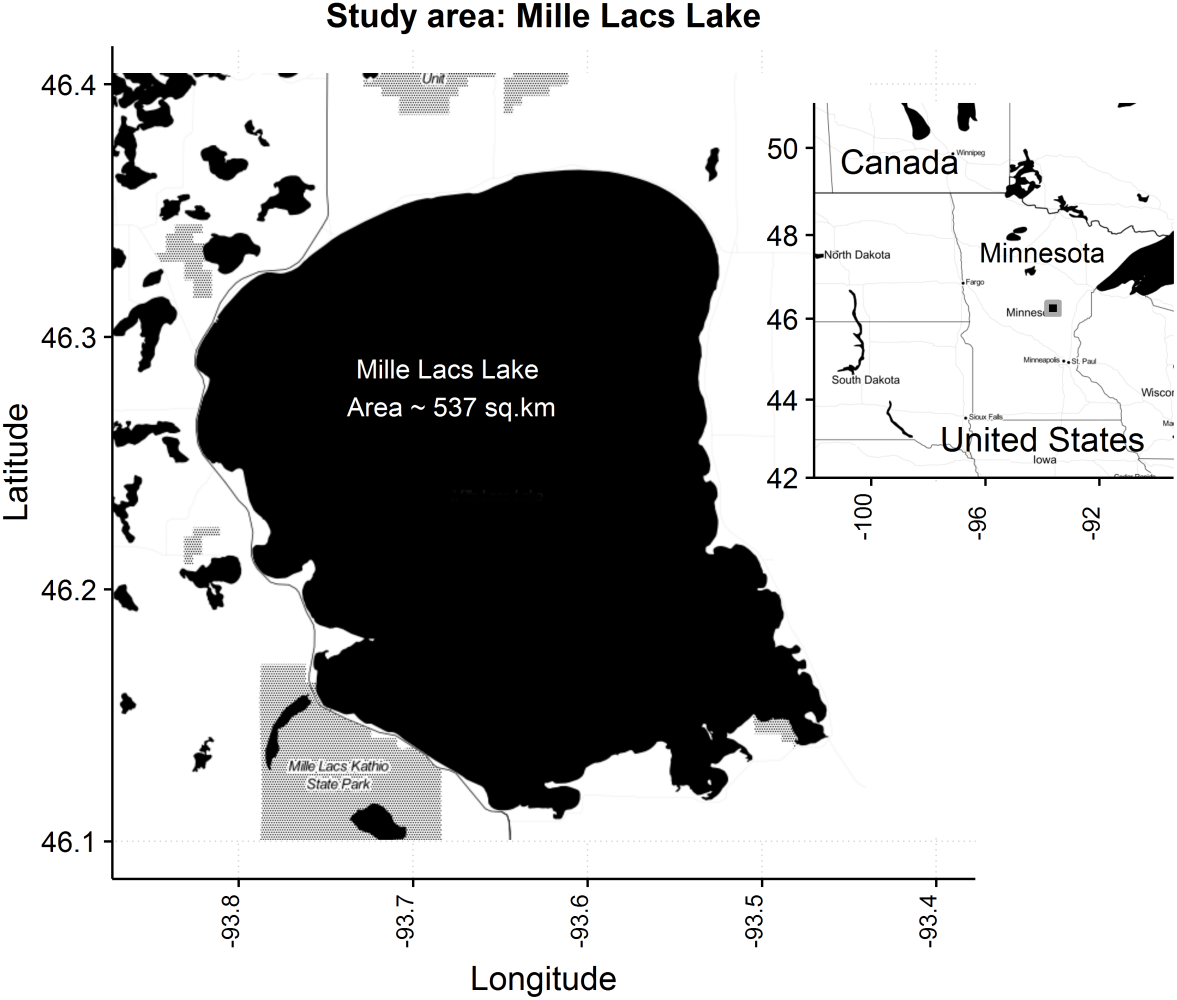
Mille Lacs Lake, Minnesota, USA. The map was generated using R package: “ggmap” [19].

Mille Lacs Lake (MLL), the second largest lake within Minnesota, is located in Aitkin, Crow Wing, and Mille Lacs counties in the east-central Minnesota (46.23°N, 93.64°W) (Fig 1). A large portion of the lake shoreline (137 km) is suitable for walleye (Sander vitreus) spawning [15], and the lake is one of the most productive large lakes for walleye fisheries in the state [16]. Yellow perch (Perca flavescens), cisco (C. artedi) different species of shiners (Notropis spp.), darters (Etheostoma spp.), and minnows (Pimephales spp.) are the main forage fish of the lake [17]. State-licensed anglers and Native American tribal (Mille Lacs Band of the Chippewa/ Ojibwe tribe) netters, spearers, and anglers are the two major resource users of the lake. Over the last three decades Mille Lacs Lake has gone through several noticeable changes such as over 90% reduction in the biomass of cisco, a cold-water stenotherm, and full establishment of zebra mussels (Dreissena polymorpha), an invasive species in the lake having potential to change the species composition of the system through their bottom-up effect on the food-web [18]. Paying attention to these changes and evaluation of their ecosystem-wide impacts are vital in sustainable management planning for the Lake.

**Fig 2:**
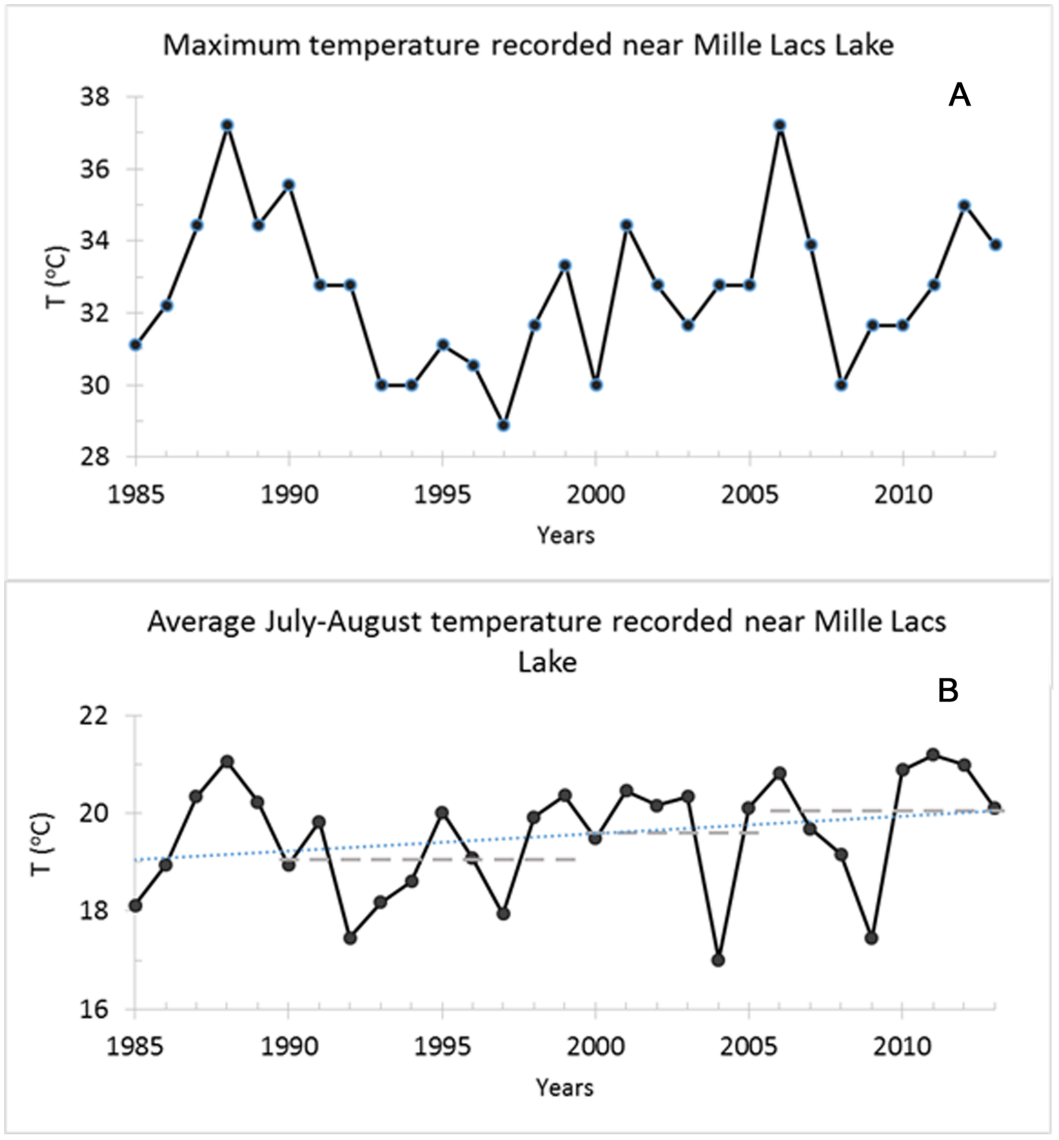
Maximum and average air temperature in July-Aug recorded at Isle station, near Mille Lacs Lake. Panel A shows maximum temperatures. Panel B shows average temperatures. In panel B the dashed grey lines show the average temperature for the corresponding time-frame (1990–1999, 2000–2005, and 2006–2013, and the blue dotted line is the trend line through the data.

An assessment of the impact of climate change on freshwater species in the USA found that “suitable thermal habitat” for cold-water species would decrease leading to a shrinkage in the distribution of these species and in-turn reduce opportunities for recreational fisheries [20]. Maximum air temperature recorded in the months of July and August near the lake fluctuated between 29°C and 37°C and was over 35°C in several years [21] (Fig 2A). The average temperatures increased by over 1°C since 1990: average temperature between 2000–2005 was 0.60°C higher than the previous decade (1990–1999), with a further increase of 0.44°C during the 2006–2013 period (Fig 2B). Mass mortalities of ciscoes, because of high temperature and low DO level in several Minnesota lakes (including Mille Lacs Lake) have been well documented [22–24]. Similarly, burbot, another cold-water stenotherm, are widespread in Minnesota, and its decline in several lakes including Mille Lacs Lake have been reported by fisheries biologists and anglers [25]. This decline in the southern reaches of its distribution is also hypothesized to be related to the level and duration of rising temperature [25, 26]. We present a study here to show an example of using an ecosystem model to explore the change in a lake ecosystem following a change in the productive capacity of cold-water species, cisco and burbot, with a change in temperature. Smallmouth bass is close to the northern edge of its native range (see Fig 1 in [14]) where it would be expected that an increase in temperature would be most beneficial. We also explore the beneficial effect of higher temperatures on smallmouth bass, especially the feeding behaviour of younger bass [14, 27] by linking the predator-prey relationship in the model to temperature pattern.

### EwE modelling: Core concepts

Our Mille Lacs Lake ecosystem model was developed using the Ecopath with Ecosim approach [28, 29]. Details of the EwE modelling suite can be explored in Christensen and Walters [29]; however, the following section presents fundamentals of the modeling routine. The Ecopath model initializes the first year of the ecosystem model based on the trophic mass-balance principle, where removals from the system (by predation, fishing, emigration etc.) equal total production. When fishing or natural mortality changes in subsequent years, the response is reflected in the biomass dynamics of the species. The two master equations of Ecopath explains the energy balance. The first equation ensures energy-balance among groups by distributing total production of a group into the catch, predators’ diet, other mortality (death caused by other than predation and catch) etc., as in (Eq 1):

$$B_{i}*{\left(P/B\right)}_{i}=Y_{i}+\sum_{j}B_{j}*{\left(Q/B\right)}_{j}*DC_{ji}+B_{i}*{\left(P/B\right)}_{i}*\left(1-EE_{i}\right)+BA_{i}+E_{i}$$ (1)

*where, subscript i and j indicates prey and predator group respectively; B stands for Biomass, P for production, Y for total fishery catch, Q for consumption, and E for net migration rate; DCji is the fraction of prey i in the diet of predator j; BA accounts for biomass accumulation; and EE explains ecotrophic efficiency i.e. fraction of group mortality explained in the model*.

The second equation explains the energy balance within a functional group as (Eq 2):

Consumption=Production+Respiration+Unassimilatedfood (2)

Mille Lacs Lake ecosystem was represented by fifty functional groups in the Ecopath model. The tropho-dynamic model represented the ecosystem state in 1985 and was fitted in Ecosim with the historic time-series data of catch and abundance over a 22-year period from 1985–2006. Ecosim is a dynamic component of EwE suite which keeps track of changes in the biomass of species (functional groups) as a function of temporal changes in their catch patterns, food-web (predators-prey interaction), and environmental conditions. The dynamic change is assessed using Eq (3) derived from the first master equation of Ecopath:

$$\frac{dB_{i}}{dt}=g_{i}\sum_{j}Q_{ji}-\sum_{j}Q_{ij}+I_{i}-\left(MO_{i}+F_{i}+e_{i}\right)*B_{i}$$ (3)

*where, dBi/dt is the biomass growth rate of group I in time-interval dt; g_i_ denotes net growth efficiency (production/consumption) of group I; Qji is the consumption by group I while Qij is for group I consumed by predators; Ii is the immigration rate; MOi explains other mortality excluding fishing and predation and Fi is the fishing mortality*.

Functional groups details and important aspects of model parameterization and temporal simulation are included (S1 Table).

### Application of temperature effect in EwE

Forcing function routines in Ecosim are used to incorporate environmental effects such as the effects of changes in temperature patterns. The shape of the function follows the changes in the given environmental variable. Temperatures in July and August of 2000–2005 were on average higher than the temperatures observed in 1985, the year Ecopath model was initialized to. To represent a regime of high temperatures in the forcing function, we used the average July-August temperatures from the year 2000 to 2005. We assumed that temperatures in the remaining months were within the tolerance limits for both species and hence did not require to be included in the forcing function. Setting up the forcing function this way allowed us to simulate the ecosystem at the higher temperature level observed in recent years.

**Fig 3:**
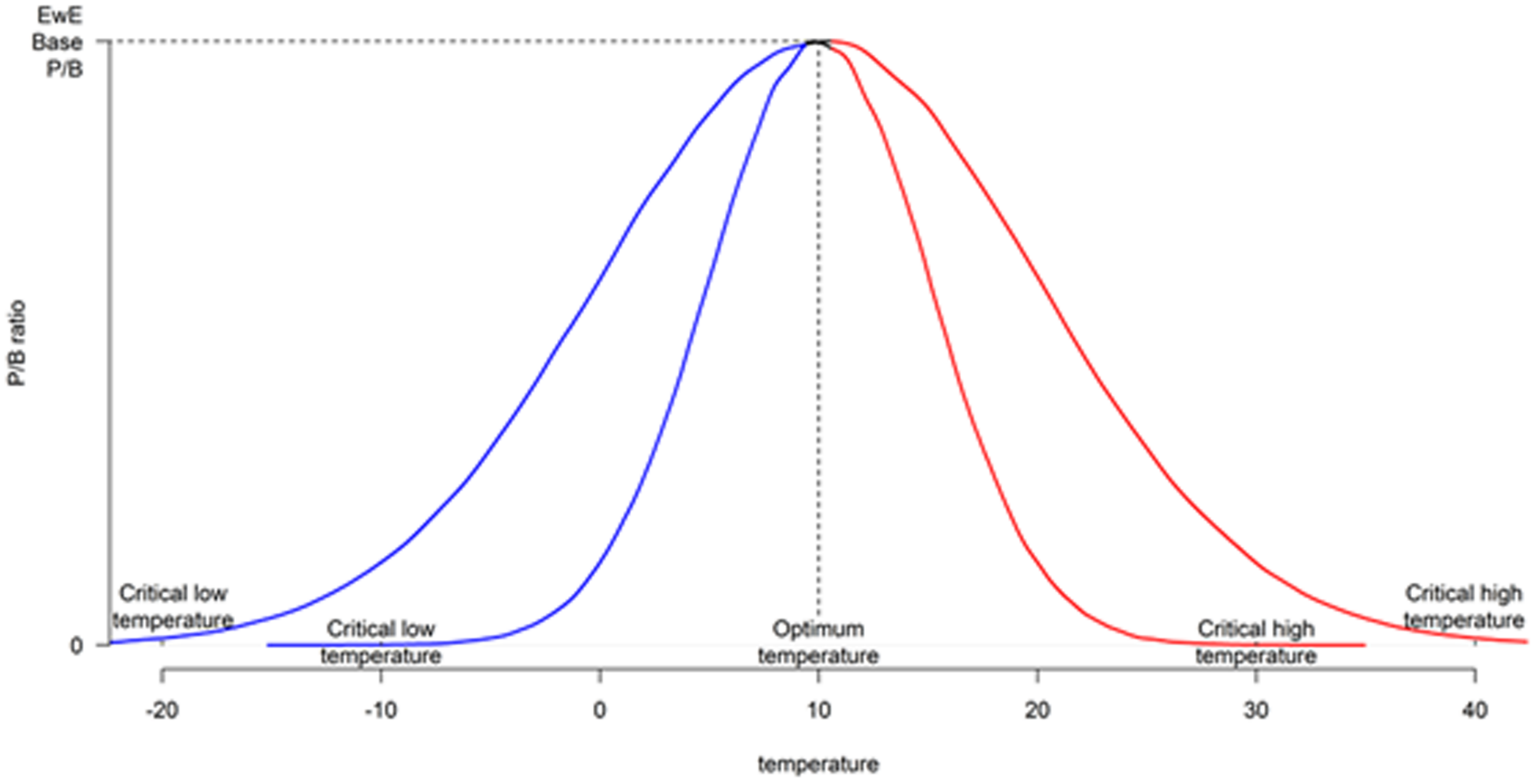
Change in P/B as temperature deviates from the optimum. The slope of the decline of P/B value depends on the difference between the optimum and the critical temperature: for a species with small tolerance range, the P/B decreases sharply compared to a species with wide tolerance rage. Also, EwE allows the user to parameterise the temperature tolerance below optimum (blue) and above optimum (red) separately (i.e. there is no restriction that the tolerance map should be symmetric around the mean).

Optimum temperature and tolerance ranges were provided for both cisco and burbot, and the temperature forcing function was applied to the tolerance ranges. For cisco and burbot, previous studies have indicated that higher temperatures decrease the suitable thermal habitat and thereby affect the productive capacity of the fish in the lake. The option available in Ecosim to model this effect is to apply the temperature forcing function on P/B ratio for the group. At the optimum temperature, the P/B of the groups are equal to their Ecopath base P/B, and any changes in temperature (increase or decrease) away from the optimum force the P/B to decline; therefore, P/B responds dynamically to the given temperature profile (forcing function). The rate at which the P/B declines is reflected by the slope of the curves—P/B declines sharply for the species with small temperature tolerance range and vice versa (Fig 3). The arms of the temperature tolerance function on the left and right of the optimum temperature are independent and are not required to be symmetric. Since the study relates to the effect of warming in summer months, we assumed that left side of the tolerance function (tolerance below optimum) was flat. The optimum temperature for cisco is below 20°C while for burbot is below 18.2°C [30].The upper tolerance level for cisco was obtained from the literatures of Edsall and Colby [31] for younger cisco and Colby and Brooke [32] for adult cisco. Younger cisco can survive higher temperature levels than adults; Edsall and Colby [31] reported an upper lethal temperature of younger cisco as 26°C. Burbot is reported to show the first response to temperature stress at temperatures 23.7°C, and temperatures above 27.5°C are found to be critical [33].

As mentioned before, the phase of high temperature has a positive influence on the growth of smallmouth bass [27] especially young smallmouth bass [14]. Optimum temperature for smallmouth bass ranges from 20 to 27°C [30]; however, the preference range was even higher in the laboratory measurements [34]. To account for the influence of temperature on feeding behaviour of young smallmouth bass, the temperature forcing function was applied on the vulnerability parameter between juvenile smallmouth bass and all its prey such that the higher temperatures were advantageous for the predator. In Ecosim, the prey population is split into ‘vulnerable’ and ‘invulnerable’ fraction, and the transfer rate between the two fractions is defined as vulnerability [29, 35, 36]. Each predator-prey trophic interaction is assigned a vulnerability (v) value, from one to infinity. If v = 1, a bottom up or donor driven relationship is implied. Assigning a high value implies a predator-driven interaction, in which predation mortality is proportional to the product of prey and predator abundance (i.e., Lotka-Volterra). This implies a high flux rate for prey species in and out of vulnerable biomass pools. For a high value for vulnerability parameter, if the predator biomass doubles, the increase in predation mortality would be approximately 2 times. If vulnerability (v = 1), a similar increase in predator biomass will not have a large effect on the predation mortality.

### Fitting the Ecosim model

The base Ecosim model with 50 functional groups was tuned with historic time-series data of abundance from 1985 to 2006 in order to reconstruct the historical trends; the process is termed model-fitting. The first step in the fitting process was to identify the most critical vulnerability interactions in the model. The sensitivity of the sum of squared differences between model predictions and time-series data points with changes in vulnerability values were analysed in Ecosim. The fitting process involved calibrating the vulnerability interactions which highly influenced and reduced the sum of squared differences between the observed and predicted time-series were selected. The fitting exercise can be performed while including the environmental forcing functions in Ecosim. When a forcing function is applied, the fitting process accounts for the change in the associated parameter (P/B or vulnerability) according to the corresponding value for the forcing function in the time series. First, we attempted to fit the model without including the temperature forcing function, then we included the temperature forcing function and examined if there was an improvement in fit following the addition of the forcing function.

There is a regular stocking program for muskellunge in the lake; hence, the resultant biomass every year is not entirely dependent on the biomass changes due to food-web and fisheries interactions. Therefore, for muskellunge, the biomass was forced using absolute biomass values. The biomass vector for cormorants and other piscivorous birds were also forced in the model. The bird population was composed of birds settled on the two islands of Mille Lacs Lake and birds migrating from other areas around the lake; their relative composition cannot be captured in the functional group dynamics of the model; except in the case where the lake experiences a constant inflow every year in which case a constant rate of immigration could be used to model the group. This was the reason behind forcing the biomass of bird functional groups in the simulation.

### Estimation of MSY and Fmsy

Though EwE has a built-infunction to estimate SSmsy and ESmsy, it lacks capabilities needed to determine uncertainties around those estimates. The function works under two different conditions depending on biomass state of non-target species [9]:

1. SSmsy: while estimating the MSY of the target species, the biomass of non-target species (i.e. rest of functional groups) were forced to be static at their base ecopath value (1985 biomass).
2. ESmsy: on the contrary to SSmsy, biomasses of non-target species were allowed to respond dynamically to changing target species biomass.

Uncertainties in the estimation may arise from a number of sources such as uncertainties in the data, input parameters, and complexity of models’ structure simulating the natural process. Monte Carlo (MC) simulation is a widely used method to assess uncertainties around the estimates [37]. In order to estimate MSYs with uncertainties, we exploited MC facilities available within the Ecosim Scenario component of EwE. We constructed a time-series extended for 500 years for each target species; the values for fishing mortality (F, yr^-1^) of the target species were incremented step by step (25 steps) and the ecosystem was allowed to equilibrate for 20 years (25*20 = 500) at each incremental F value. The F for the non-target species were held at their base ecopath value (i.e., unchanged). The F value that resulted in maximum yield was recorded as Fmsy and the corresponding yield as MSY. This method was applied to estimating ESmsy under the temperature forcing function. In summary, we estimated the reference points with and without including the temperature forcing function, and this allowed us to estimate how much the lake ecosystem and the fisheries reference points were affected by the changes in the temperature pattern.

Sensitivity analysis: Sensitivity of the results to the Ecopath input parameters (B, P/B, Q/B, EE) was explored. For each run, MC simulation randomly selected a set of parameters within a 10% coefficient of variation (CV = 0.1) for the B, P/B, Q/B, EE parameters. Some sets of parameters did not satisfy Ecopath mass-balance (EE, the fraction of production of functional groups that goes to their predator’s diet or fished out from the system, exceeds 1.0 plus some tolerance); in that case, the trial was considered unsuccessful, and the MC routine drew another set of parameters values. We present results from 80 successful-runs of Ecosim MC simulations for each MSY estimation.

For clarity, the following abbreviations were used to refer different versions of MSYs in the manuscript:

1. SSmsy for single-species MSY without temperature forcing function,
2. SSmsyT for single-species MSY with temperature forcing function,
3. ESmsy for ecosystem MSY without temperature forcing function, and
4. ESmsyT for ecosystem MSY with temperature forcing.

## Results

### Fitting the Ecosim model

The Ecosim model was able to reasonably predict (S1 Fig) the dynamics of the walleye and northern pike population. The model reproduced the decline of cisco and burbot biomass observed over the time period. The model predicted an increase in yellow perch and smallmouth bass population in the lake but not to the levels indicated in the observed CPUE trends. The model predicted an increase in rock bass but a decrease in white sucker and black crappie. The period of cisco decline also saw the increase of yellow perch which became the most important forage species in the lake. The temperature forcing functions described in the previous section were important to capture the pattern of decline in cisco and burbot and the pattern of increase in smallmouth bass. The EwE sum of squares estimate improved from 173.7 to 153.7 when the temperature forcing function was included in the model.

### Trophic and temperature effects on MSY

**Fig 4:**
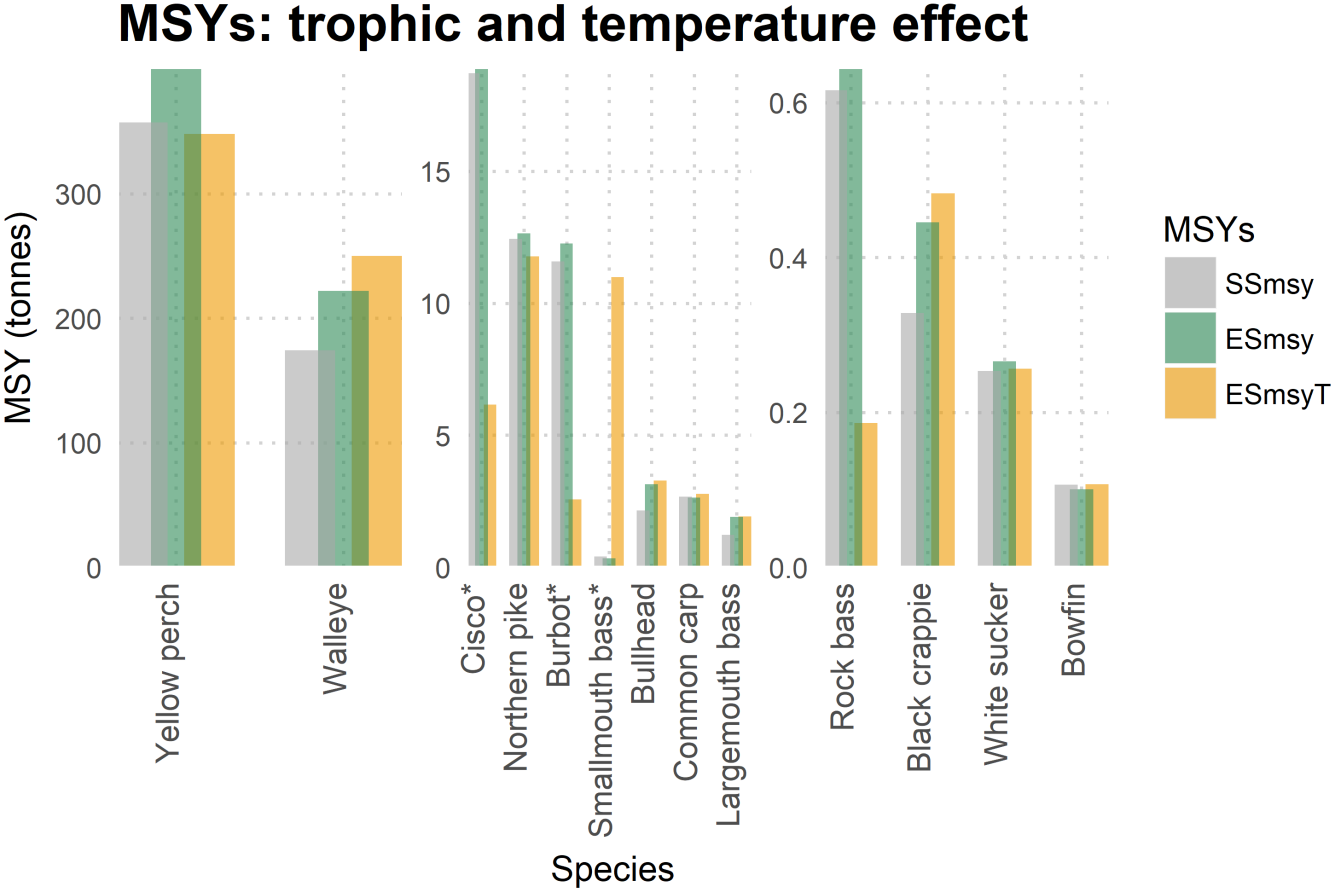
Single species and ecosystem MSY estimates. The figure shows SSmsy, ESmsy, and ESmsyT estimates for 13 species in Mille Lacs Lake. * indicates species on which the temperature forcing function was applied.

SSmsy and ESmsy for 13 species with and without the temperature forcing function were estimated (Table 1, Fig 4). When temperature forcing was not included, the ESmsy was nearly equal or higher than the SSmsy for all the 13 species (Fig 4). Implementation of temperature directly influenced the ESmsyT estimates for cisco, burbot, and smallmouth bass and indirectly influenced (trophic cascade) the estimates for several other species—the ESmsyT estimates were lower than that of ESmsy as well as SSmsy for many species such as yellow perch, northern pike, and rock bass (Fig 4).

**Table 1:** SSmsy and ESmsy for 13 species in Mille Lacs Lake with and without the temperature forcing function.

Species	With temperature forcing function				Without temperature forcing function			
	Single-species		Ecosystem		Single-species		Ecosystem	
	MSY	Fmsy	MSY	Fmsy	MSY	Fmsy	MSY	Fmsy
Walleye	170.25	0.22	249.80	0.28	174.27	0.21	221.77	0.27
Yellow perch	336.86	1.08	347.82	2.87	357.11	1.23	399.93	2.86
Northern pike	12.38	0.13	11.77	0.14	12.43	0.14	12.65	0.14
Cisco	11.46	0.22	6.17	0.13	18.71	0.27	18.86	0.27
Smallmouth bass	14.87	0.08	10.99	0.09	0.41	0.07	0.34	0.07
Rock bass	0.48	0.06	0.19	0.05	0.62	0.06	0.64	0.07
White sucker	0.25	0.07	0.26	0.07	0.25	0.07	0.27	0.07
Black crappie	0.17	0.02	0.48	0.04	0.33	0.03	0.45	0.04
Burbot	4.04	0.03	2.56	0.02	11.59	0.04	12.25	0.04
Common carp	2.70	0.07	2.77	0.07	2.67	0.06	2.63	0.06
Largemouth bass	1.15	0.05	1.91	0.07	1.23	0.05	1.89	0.08
Bowfin	0.10	0.03	0.11	0.03	0.11	0.03	0.10	0.03
Bullhead	2.16	0.08	3.27	0.11	2.15	0.07	3.14	0.10

The key reason for obtaining higher ESmsy corresponding to SSmsy was a consequence of gradual increases in the availability of the prey to the target species as the target species was fished down; the biomass of the target species decreased, and this released predation pressure from their prey. We also noticed several other trophic effects propagating through ecosystem: in some cases, trophic interactions enhanced compensation in the targeted species while in others trophic interactions weakened the compensation.

**Fig 5:**
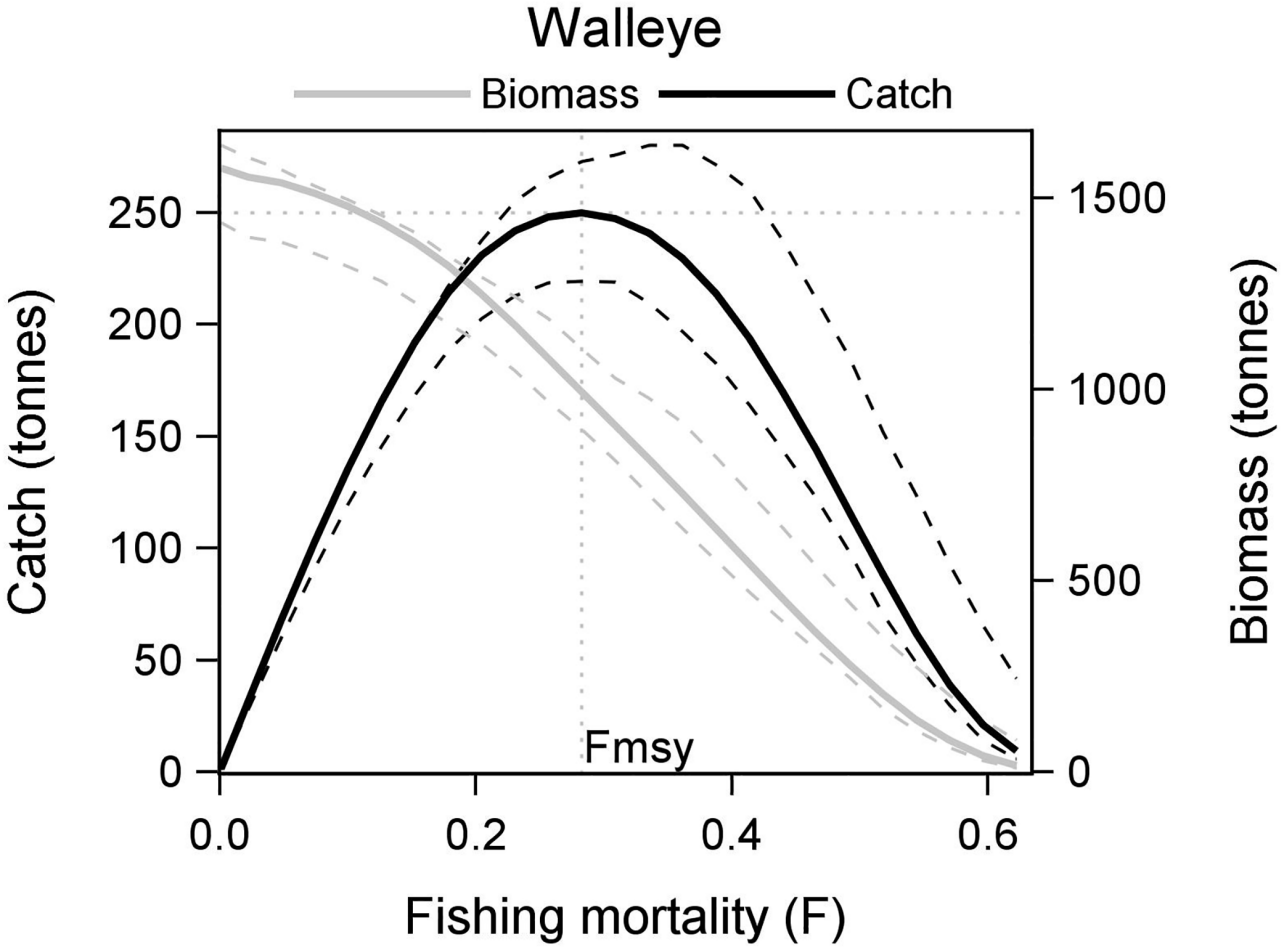
Temperature-influenced ecosystem MSY (ESmsyT) for walleye in Mille Lacs Lake. The black line (solid) shows predicted catch, and grey line (solid) shows predicted biomass. The black and grey dashed lines show the interquartile range for catch and biomass respectively.

**Fig 6:**
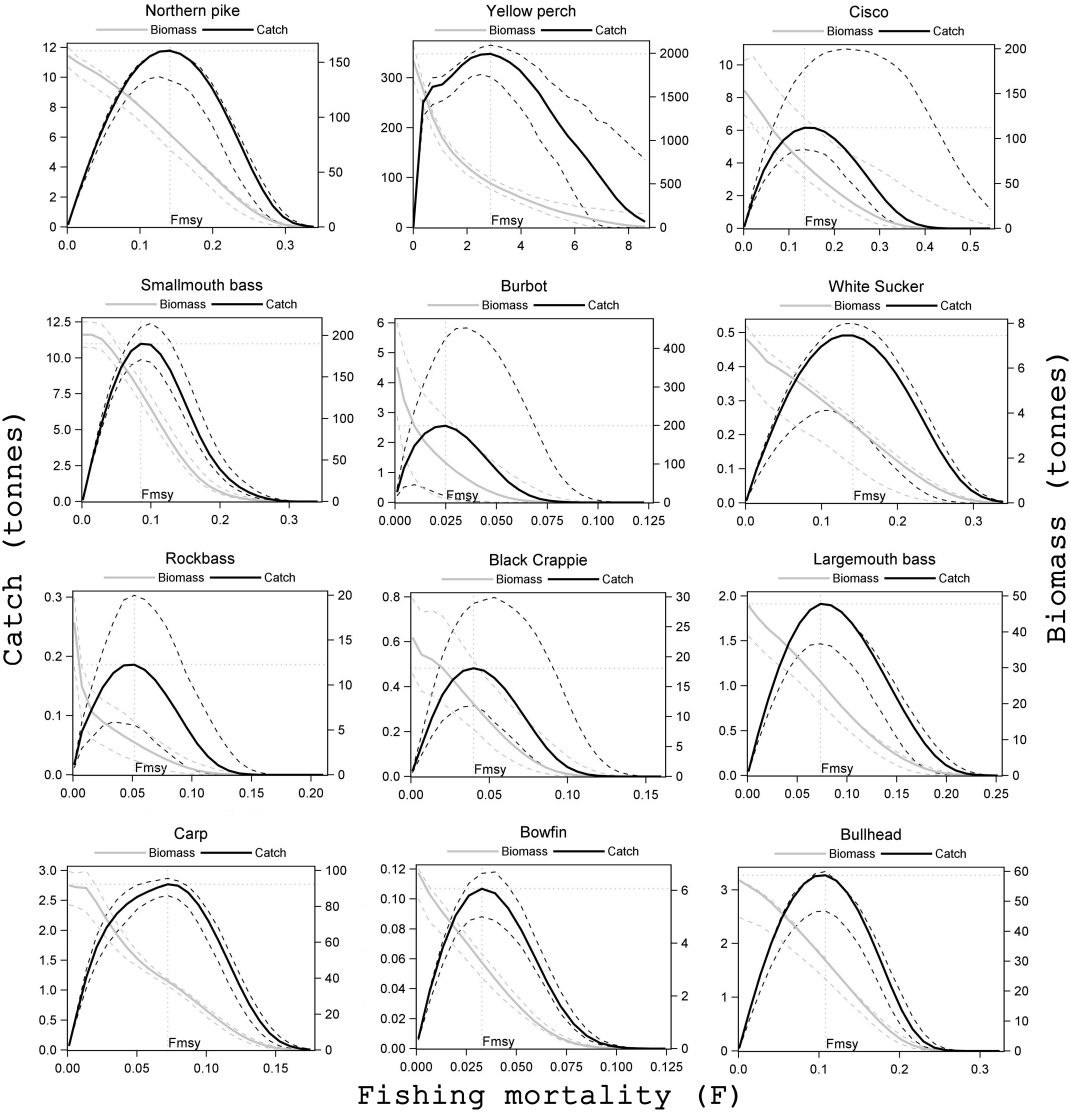
Temperature-influenced ecosystem MSY (ESmsyT) for key recreational species in Mille Lacs Lake. The black line (solid) shows predicted catch, and grey line (solid) shows predicted biomass. The black and grey dashed lines show the interquartile range for catch and biomass respectively. Species included are northern pike, yellow perch, cisco, smallmouth bass, burbot, white sucker, rock bass, black crappie, largemouth bass, carp, bowfin, and bullhead.

In the following section, we first describe the differences in the estimations because of trophic effects, i,e. between SSmsy and ESmsy; then we describe the differences due to the combined effect of trophic and temperature, i, e. between SSmsy and ESmsyT. The sensitivity analyses are presented as the lower and upper grey dashed MSY curves (Figs 5 and 6). A summary of all types of MSYs estimates are in Table 1, and species-specific figures depicting the temperature-influenced ecosystem MSY estimates (ESmsyT) with uncertainties and Fmsy for all the species were provided in Figs 5 and 6. For most species in Fig 6 (except rock bass), biomass in the Ecopath model was estimated by fixing the EE values and therefore there is higher underlying uncertainty for these species than for results presented in earlier figures (Figs 5 and 6).

#### Walleye

The ESmsy estimate of walleye was higher than the SSmsy (Fig 4). The higher ESmsy was supported by an increase in its prey such as cisco, shiners and minnows, and hex mayflies. Yellow perch was the major prey of the species, and we expected an increase in its abundance as F was increased on walleye. But that was not the case; a decline in yellow perch was observed mainly because of increased predation from burbot and northern pike. This was a case of indirect food web influence observed in the ecosystem model. When walleye abundance decreased, predation pressure on shiners and minnows was reduced; trace amounts of predation on young burbot weakened. There was a small increase in the population of a competitor species of walleye—burbot, and this change caused a decline in yellow perch population. When the temperature forcing was implemented, the response of increasing fishing pressure on walleye was very similar except for a better performance of shiners and minnows, which probably contributed to a higher ESmsyT (~43%) estimate for walleye (Fig 5).

#### Northern pike

Fishing was a major component of mortality on northern pike; thus, the ESmsy estimated was merely 2% higher than the SSmsy. This small advantage was probably due to an increase in prey species such as rock bass and yellow perch.

The combined trophic and temperature effect resulted a slightly lower (5%) ESmsyT estimation (Fig 4) due to the lower performance of yellow perch, rock bass and temperature influenced decline in cisco. Northern pike caused some predatory pressure on younger walleye; therefore, the walleye population showed a minor improvement when the biomass of northern pike declined slightly.

#### Yellow perch

Yellow perch is the most important prey of the Mille Lacs ecosystem; it constitutes a major portion in the diet of almost all the piscivores. Much differences were not observed between the SSmsy and ESmsy estimates for yellow perch (Fig 4). A decline in yellow perch was observed following an increase in fishing pressure. Following this decline, predators northern pike and walleye decreased and prey such as darters, pelecypods, crayfish, and gastropods improved. However, compensation from the release of pressure on prey species was not available for yellow perch as several predator species such as walleye, northern pike, and smallmouth bass utilized the prey species. After an initial decline, northern pike stabilized in the later years of simulation as the availability of crayfish and darters increased in the system. Improved biomass trends of smallmouth bass and cisco were also observed; the healthier population of crayfish contributed to the former while pelecypods and zooplankton to the latter. We also expected a reduction in the abundance of fish-eating birds such as cormorants as yellow perch contributed to the diet of these birds, but an augmented cisco and smallmouth bass balanced the loss of yellow perch. We also observed an instability in yellow perch dynamics when fishing pressure was increased. This instability resulted in a MSY curve which was relatively flat over a wide range of F values (Fig 6, “yellow perch”). Thus, we found that it was very difficult to determine the SSmsy and ESmsy estimates for yellow perch.

#### Cisco

For cisco, the trophic only effect did not make much difference to the estimates; therefore, ESmsy and SSmsy were very close (Fig 4). Cisco biomass declined in response to a gradual rise in F, which triggered an increase in the biomass of prey such as zooplankton, pelecypods, and gastropods and a decline in predator northern pike. Both effects would have been conducive for a higher ESmsy estimate for cisco if the decline of burbot and northern pike had not triggered yellow perch to increase. The rise in yellow perch added extra predatory pressure on zooplankton, which constituted a large percentage of cisco diet (a food competition between cisco and yellow perch). These positive and negative effects counterbalanced each other, and ESmsy for cisco was not altered by these compensatory responses. Since yellow perch is the most important prey of walleye, walleye was not affected by the decline of cisco.

On the other hand, the effect of temperature caused cisco to decline excessively (biomass was nearly one-third of the SSmsy or ESmsy level). We also estimated the temperature-influenced single-species MSY (SSmsyT) for cisco and found the estimate was within the range of the estimates from the state-space modelling in Kumar et al. [24]. Compared to SSmsyT, the ESmsyT was lower due to the lesser compensation from pelecypods (a prey of yellow perch and cisco) and crayfish (a prey of yellow perch and smallmouth bass). The confidence interval around ESmsyT varied widely between 5 to 11 tonnes for cisco (Fig 6).

#### Smallmouth bass

For smallmouth bass, the ESmsy was 16% lower than SSmsy (Fig 4); the decrease was mostly due to the competition for prey such as black crappie and crayfish from other predators such as northern pike and yellow perch. However, the direct effect of temperature on the vulnerability of smallmouth bass to its prey could be easily seen in the ESmsyT estimate, which was over 32 times higher than the ESmsy. Getting higher abundance as in the lake gillnet surveys in recent years supports this conclusion. The temperature forcing applied was the same as in the fitted model where vulnerability value in the predator-prey relationship changes in direct proportion to temperature change. The results here show that this hypothesis can explain the recent boom in smallmouth population in the lake. However, it needs to be explored how the results would vary if the relationship between vulnerability and temperature forcing function was non-linear. The sensitivity analysis performed showed that the ESmsy estimate would range between 9 and 12.5 tonnes (Fig 6); while this sensitivity analysis includes uncertainty on P/B, Q/B, and EE parameters in EwE, it does not include sensitivity on the vulnerability parameter or the relation between the vulnerability and temperature forcing function.

#### Burbot and white sucker

Trophic compensation from its prey such as juvenile yellow perch and crayfish caused a slightly higher (6%) ecosystem MSY estimate for burbot. Incorporating temperature negatively affected burbot, resulting a nearly one-fifth decline in ESmsyT (Fig 4); the confidence interval ranged between 0.6 to 5.8 tonnes (Fig 6).

For white sucker, we obtained very similar estimates across all the cases of MSY estimations—differences were no more than 5% (Fig 4). Burbot and white sucker equally contributed (17% each) in the diet of otters and minks. The decline of burbot under temperature forcing condition and fishing white sucker at MSY led to a decrease in the biomass of otters and minks. The decrease in otters and minks also favored cormorants and other piscivorous birds.

#### Rock bass

For rock bass, the SSmsy and ESmsy estimates were very similar (Fig 4). Crayfish is a major item in the diet of the both smallmouth and rock bass. Smallmouth bass population competed with rock bass for crayfish resulting in very similar SSmsy and ESmsy estimates for rock bass.

#### Bullhead and largemouth bass

The higher ESmsy estimates for Bullhead and Largemouth bass were the result of direct prey-predator interactions. Bullhead benefitted from invertebrates and decrease in biomass of its main predator, largemouth bass. Largemouth bass was benefitted from an increase in its prey, bullhead. Not much difference was observed between ESmsy and ESmsyT estimates for bullhead and largemouth bass (Fig 4).

#### Other species: Bowfin, black crappie, and common carp

The ESmsy for Bowfin was slightly lower (6%) than SSmsy. Bowfin predates on black crappie and on trace amounts of largemouth bass along with other prey. When bowfin was fished heavily, a small improvement in largemouth bass was observed, but this had a large negative impact on black crappie. The reduction in black crappie resulted in a lower ESmsy for Bowfin. Under temperature forcing, the negative impacts were not as strong and the ESmsyT for bowfin was slightly higher compared to ESmsy. For black crappie, the ESmsy was greater than the SSmsy due to losses in its predators’ abundance such as largemouth bass, northern pike, bowfin and smallmouth bass. The ESmsy and ESmsyT estimates for black crappie were very similar. For common carps, there was less than 5% difference between the different MSY estimations.

### Sensitivity analysis

The sensitivity analysis allowed four of every functional group parameters in the 50 functional group model to vary within the Monte Carlo routine. We believe our results are quite encouraging. Fig 6 shows that there were several cases where the inter-quartile limit varied by several tonnes, but the uniformity in species response under the different simulations points to model stability. The MSY estimates were highly sensitive to uncertainty in Ecopath parameters for cisco and burbot, mainly because the temperature forcing function was applied to the P/B ratio and the sensitivity analysis allowed for uncertainty in this key EwE parameter.

## Discussion

### Trophic and temperature effects on MSY

In many cases, especially when temperature forcing was not in effect, the SSmsy estimates were more conservative than the ESmsy estimates. When the temperature effects were accounted for, the model predicted a mixed response (Fig 4, Table 1): for some species, the ESmsy was larger and for others, it was smaller. The MSY estimates for cisco and burbot were lower when the temperature effects were considered. For smallmouth bass, temperature effects led to higher MSY estimates. However, indirect effects on the estimates were also observed in several other species. For example, increased competition with smallmouth bass for crayfish led to lower MSY estimates for rock bass and yellow perch. Similarly, the lower performance of cisco and yellow perch led to a lower MSY estimate for northern pike.

In a similar analysis, Walters et al. [9] found that ecosystem MSY estimates increased as a result of compensation from predator and/or prey species. Similar results favoring higher effort levels under multispecies calculations were found in Gislason [10] and Jacobsen et al. [38] mainly through adjustments in competition, predation, and cannibalism. Though it is not always easy to identify all the direct or indirect trophic interaction responsible for such compensation, this analysis identified several major linkages in the system and showed how these linkages influenced the reference point estimates. Our results showed that the compensatory response of prey influenced the ecosystem MSY estimates; therefore, exploitation of prey species needs to take into account the contribution of the prey to the predator diets. This kind of information would not be available from a single-species based analysis. Global analysis of the contribution of prey species to fisheries found that in addition to direct benefits from the catch, the total value generated by prey species was much higher due to their contribution to catch from higher trophic groups [39, 40]. It is also possible that, based on the similarity of trophic function, forage fish may be managed as a pool of species [41], but this could be problematic in a situation where differences exist between the forage species with respect to how they respond to environmental changes (for example, in Mille Lacs Lake it would not be appropriate to group cisco with yellow perch).

As mentioned, yellow perch is the leading prey species in the lake and therefore the explanation of food-web dynamics of the lake without understanding perch dynamics is inconceivable. The major factors regulating its dynamics were walleye and cannibalism within the yellow perch population. When walleye was under-exploited, yellow perch was controlled by direct predatory pressure from walleye. However, the effects were not always straightforward: when walleye biomass was reduced by fishing at MSY, contrary to expectations, yellow perch biomass declined due to the increased predation from species which were released from walleye predation. Direct compensation was not observed indicating that walleye-perch relationship is highly complicated [42]; walleye exerts predation pressure on yellow perch but also controls the other predators of yellow perch. Further, cannibalism in yellow perch resulted in instability during MSY estimations. This was manifest as the relatively flat top of the MSY curve. At increased fishing pressure, different age groups in the yellow perch population responded differently. The root cause for this response was that increase in fishing led to decrease in cannibalism. Yellow perch dynamics was also affected by smallmouth bass abundance. Increase in smallmouth bass due to temperature caused increased pressure on crayfish population, which in turn was not able to provide the same level of compensation to yellow perch population when the temperature rise was in effect, and therefore not much difference was observed between SSmsy and ESmsyT estimates for yellow perch (Fig 4).

This analysis enhances the understanding of interspecies interaction in the system and provides reference points which could serve to improve the current management plan that is largely based on single-species studies. Previous to this analysis, reference points for catch/stock status for Mille Lacs Lake were limited to a few species only, such as walleye [43], northern pike [44] and yellow perch [45]. This analysis has laid out the reference limits for more than a dozen key species of the lake, a contribution that could serve as a strategic step in the implementation of EBM [12, 46].

Our approaches allow the integration of temperature and trophic effects. We were able to identify sources of ecosystem pressure that caused lower ESmsy for cisco, rock bass and other species under temperature forcing. Temperature has a strong influence on the decline of cisco [22]. Kumar et al. [24] estimated the time-varying MSY for cisco and found that temperature accounted for nearly 36% of the change in the cisco population in Mille Lacs Lake by correlating a “temperature anomaly” with its carrying capacity in the state-space model. We obtained a very similar result for cisco from the EwE based analysis when only a temperature effect was considered. Moreover, the analysis here suggested that in addition to the direct effects of temperature, cisco also faced indirect effects of temperature rise in the form of stronger competition from yellow perch and smallmouth bass, leading to lower estimates of the limit reference points for cisco, a nearly 45% reduction in the ESmsyT estimate.

Several studies reported instances where an increase in temperature favored smallmouth bass [47]. The present study showed that projected increase of productivity of smallmouth bass would create competition for crayfish in Mille Lacs Lake. This competition for crayfish could negatively affect rock bass, cisco, and yellow perch. However, temperature rise also led to the decline of burbot, which is predatory on yellow perch; this released some predation pressure from yellow perch. The analysis here, therefore, lays out several hypotheses of how the ecosystem would respond to changing temperature. Moreover, the analysis re-emphasizes that estimates of reference points could change with environmental changes, and it is important to re-estimate the reference points when it is expected that such changes could affect the productivity of a species.

### Sensitivity analysis

In our paper, we assumed that P/B ratios followed the temperature tolerance curve (Fig 3). The sensitivity of cisco and burbot MSY estimates to P/B ratios shows that it is important to explore the shape of this function in more depth. Similarly, the influence of the temperature forcing function on smallmouth bass is defined solely based on obtaining a better fit in the Ecosim model by using this function. We assumed that vulnerability of prey to smallmouth bass varied in direct proportion to temperature. Other alternate functional forms to describe this relationship were not explored. This aspect also needs a more careful directed study.

The diet matrix parameters help in calculating the mass-balance constraint of the functional groups in Ecopath, and the Monte Carlo analysis can be criticized for not allowing the diet matrix parameters to vary. However, the strength of the predatory-prey relationship (i.e. predation mortality estimates within the model) and the consumption varies depending on the biomass, P/B, and Q/B values of the predator and the prey species. Therefore, all the important predator-prey linkages will be affected by the Monte Carlo routine on the 4*n (number of functional groups) parameters in the model (~200 parameters in the model). Further, adding Monte Carlo on diet preferences has not yet been tested within the EwE software on account of the complexity involved. In this Mille Lacs Lake EwE model, which attempts to model 50 functional groups, there are more than 2,000 diet interactions. Allowing a sensitivity analysis on each of these interactions, while constraining for mass-balance, will be a highly computationally intensive task (because of the number and complexity of interactions among parameters to achieve mass balance for each Monte Carlo choice).

In terms of structural uncertainty, a direct temperature effect was included for three species, but it might be possible that temperature had direct effects on other species too that we did not explore. So, there is a scope to investigate other effects of temperature in future. Moreover, other sources of structural uncertainty related to spatial complexity [48] could be explored through spatial extensions of the model using Ecospace.

## Conclusions

When temperature effects were incorporated, the SSmsyT estimates for cisco and burbot were much lower than when temperature effects were not included (SSmsy). We found that the SSmsyT estimates were similar to the values obtained from the surplus production modelling [24]. However, the ESmsyT estimates were lower indicating that there were ecosystem pressures on cisco and burbot that exerted further pressure on these populations thus exacerbating the effect of temperature change.

Several modelling studies have explored the influence of environmental changes on dynamics of individual species [49, 50], and some have explored ecosystem-wide impacts [51–53]. A study that explored how planktons and fish populations would respond to simultaneous effects of fishing and change in upwelling conditions found that the response of forage fish was key to understanding the ecosystem-wide effect of the different drivers [52]. Similarly, bottom-up effects of El Nino was felt primarily by plankton groups and secondary consumers and then these effects cascaded up to the higher trophic levels of the ecosystem [53]. We found important bottom-up effects transferred from yellow perch to the dynamics of most other fish species in the lake. Yellow perch emerged as the single most important forage fish in the lake after the decline of cisco. The other important forage species were crayfish, darters, and so on. Changes in the ecosystem that resulted in an increase in the biomass of forage species were compensatory for the predator species, and vice versa, the interactions that caused a decline in forage species biomass resulted in a decline in the predator species. Our results suggest that taking into account ecosystem considerations were most important when the prey species in the ecosystem have a commercial fishery on them, for example, fishing yellow perch at Fmsy can lead to a decline in biomass of important recreational species like walleye, northern pike, and burbot. In the case of smallmouth bass, we found that when the ESmsy was lower than the SSmsy, it was mostly a result of competition for prey, but when the increase in temperature was incorporated in the calculation, the ESmsyT estimate increased many fold. Mackinson et al. [51] found that expected compensation was sometimes delayed or diminished due to “trade-offs in competition and predation interactions”. Overall the paper provided preliminary indications that when there is fishing on prey species, the recovery of over-exploited predator groups might be difficult. Adding the effect of environmental drivers changed the reference point estimates for several species. Therefore, it is important for management to re-evaluate the reference points when it is expected that there could be a change in species productivity either due to changes in the environmental condition or due to major shifts in community structure or relative abundance of species.

## Supporting information

S1 Table Parameterization of Mille Lacs Lake ecosystem model. (PDF)

S1 Fig Ecosim model fitting from 1985 to 2006. The dots are the observations and the lines are the Ecosim predictions. Prediction lines that exactly follow the dots are forced biomasses.
(TIF)
