# Supplementary material for: Ecosystem approach to fisheries: Exploring environmental and trophic effects on Maximum Sustainable Yield (MSY) reference point estimates
Source: PLoS One. 2017 Sep 28;12(9):e0185575. doi: 10.1371/journal.pone.0185575 (PMC5619794; doi:10.1371/journal.pone.0185575)
Supplement: S1 Table — (PDF) [file pone.0185575.s001.pdf]

S1 Table. Parameterization of Mille Lacs Lake ecosystem model.

Ecopath functional groups and their parameters: values and data sources. Abbreviation: B=Biomass, P=Production, Q=Consumption, EE=Ecotrophic Efficiency, ME=Model estimates (value in bold represents the estimation by the model).Note: the diet information for some cases in the model was adjusted from their actual sources at the diet matrix workshop with Minnesota Department of Natural Resource (MNDNR) staff at Aitkin, Minnesota and during the Ecopath balancing process.

SN	Functional groups and parameters	Value	Source of data and general comments
**1**	**Walleye (*Sander vitreus*) age 0**		
	B	**0.003**	ME for stanza [1]
	P/B	1.000	Adjusted during the model balancing
	Q/B	**17.172**	ME for stanza [1]
	EE	**0.800**	ME based on mass-balance principle
	Major preys	5,19,28,30	Gut-content analysis
**2**	**Walleye age 1**		
	B	**0.053**	ME for stanza [1]
	P/B	0.600	Adjusted during the model balancing
	Q/B	**6.993**	ME for stanza [1]
	EE	**0.94**	ME based on mass-balance principle
	Major preys	5,28,32,6,13	Gut-content analysis
**3**	**Walleye age 2**		
	B	**0.149**	ME for stanza [1]
	P/B	0.400	Adjusted during the model balancing
	Q/B	**4.337**	ME for stanza [1]
	EE	**0.640**	ME based on mass-balance principle
	Major preys	5,28,6,39,13	Gut-content analysis
**4**	**Walleye age 3+**		
	B	2.496	Drake [2]
	P/B	0.303	Drake [2]
	Q/B	2.284	Estimated by an empirical formula [3]
	EE	0.259	ME based on mass-balance principle
	K	0.240	Fitting of von Bertalanffy Growth Function (VBGF) to age-length key obtained from MLL survey data
	Major preys	5,28,6,39,14,15	Gut-content analysis
**5**	**Yellow perch (*Perca flavescens*) age 0**		
	B	**0.952**	ME for stanza [1]
	P/B	5.000	Adjusted during the model balancing
	Q/B	**15.047**	ME for stanza [1]
	EE	**0.908**	ME based on mass-balance principle
	Major preys	46	Gut-content analysis
**6**	**Yellow perch age 1**		
	B	**0.676**	ME for stanza [1]

	P/B	2.100	Adjusted during the model balancing
	Q/B	**6.036**	ME for stanza [1]
	EE	**0.819**	ME based on mass-balance principle
	Major preys	39,38,43,41,5,40	Based on gut-content analysis and study by Tarby [4]
**7**	**Yellow perch age 2+**		
	B	1.350	Surplus production model (SPM) using time-series of catch and assessment gill net CPUE data of the lake [5]
	P/B	0.783	M estimated by an empirical equation [6]; F=C/B; and F+M=Z=P/B [7]
	Q/B	3.378	Estimated by an empirical formula [3]
	EE	**0.688**	ME based on mass-balance principle
	K	**0.37**	Fitting of von Bertalanffy Growth Function (VBGF) to age length key obtained from MLL survey data
	Major preys	5,38,30,43	Based on gut-content analysis and study of Tarby [4]
**8**	**Northern pike (*Esox Lucius*) age 0**		
	B	**0.000**	ME for stanza [1]
	P/B	1.000	Adjusted during the model balancing
	Q/B	**15.492**	ME for stanza [1]
	EE	**0.740**	ME based on mass-balance principle
	Major preys	5,46,39,30,38,13	Gut-content analysis
**9**	**Northern pike age 1**		
	B	**0.003**	ME for stanza [1]
	P/B	0.400	Adjusted during the model balancing
	Q/B	**6.383**	ME for stanza [1]
	EE	**0.887**	ME based on mass-balance principle
	Major preys	7,5,6,2,32,19,21,15,14	Gut-content analysis
**10**	**Northern pike age 2+**		
	B	0.195	SPM using time-series of catch and assessment gill net CPUE data of the lake [5]
	P/B	0.210	M estimated by an empirical equation [6]; F=C/B; and F+M=Z=P/B [7]
	Q/B	2.322	Estimated by an empirical formula [3]
	EE	**0.552**	ME based on mass-balance principle
	K	0.30	Fitting of von Bertalanffy Growth Function (VBGF) to age length key obtained from MLL survey data
	Major preys	7,32,19,2,15,28,14,3,4,5,6	Gut-content analysis
**11**	**Muskellunge (*Esox masquinong*y) age 0**		
	B	**0.000**	ME for stanza [1]
	P/B	0.810	Adjusted during the model balancing
	Q/B	**11.732**	ME for stanza [1]
	EE	**0.357**	ME based on mass-balance principle
	Major preys	5,46,20,39,38,30,13	Adjusted based on study of Hourston [8]

**12**	**Muskellunge age 1+**		
	B	0.001	"Muske life table" data sheet (Tom Jones, MNDNR, pers. Comm.
	P/B	0.344	Assumed equivalent to M which was estimated using Pauly's formula [6]
	Q/B	2.110	Estimated by an empirical formula [3]
	EE	0.222	ME based on mass-balance principle
	K	0.32	Fitting of von Bertalanffy Growth Function (VBGF) to age length key obtained from MLL survey data
	Major preys	7,20,15,17,24,14,30	Based on study of Hourston [8]
**13**	**Cisco (*Coregonus artedi*) age 0**		
	B	**0.038**	ME for stanza [1]
	P/B	2.000	Adjusted during the model balancing
	Q/B	**12.001**	ME for stanza [1]
	EE	**0.890**	ME based on mass-balance principle
	Major preys	46,40	Engel [9]
**14**	**Cisco age 1**		
	B	**0.095**	ME for stanza [1]
	P/B	1.000	Adjusted during the model balancing
	Q/B	**6.508**	ME for stanza [1]
	EE	**0.853**	ME based on mass-balance principle
	Major preys	46,40,39,41,43,44	MNDNR approximation
**15**	**Cisco age 2+**		
	B	0.237	Estimated from sampling data [10]
	P/B	0.650	M estimated by an empirical equation [6]; F=C/B; and F+M=Z=P/B [7]
	Q/B	4.622	Estimated by an empirical formula [3]
	EE	**0.608**	ME based on mass-balance principle
	K	0.72	Fitting of von Bertalanffy Growth Function (VBGF) to age length key obtained from MLL survey data
	Major preys	46,40,39,41,43,44	MNDNR approximation
**16**	**Smallmouth bass (*Micropterus dolomieui*) age 0**		
	B	**0.000**	ME for stanza [1]
	P/B	1.500	Adjusted during the model balancing
	Q/B	**11.869**	ME for stanza [1]
	EE	**0.912**	
	Major preys	30,21,29,40,41	From Pelham *et al.* [11]
**17**	**Smallmouth bass age 1+**		
	B	0.020	Guesstimate
	P/B	0.530	M estimated by an empirical equation [6]; F=C/B; and F+M=Z=P/B [7]
	Q/B	2.765	Estimated by an empirical formula [3]
	EE	**0.179**	ME based on mass-balance principle
	K		

	Major preys	38,29,41,5,6	From Frey *et al.* [12]
**18**	**Rock bass (*Ambloplites rupestris*)**		
	B	0.022	SPM using time-series of catch and assessment gill net CPUE data of the lake [5]
	P/B	0.430	M estimated by an empirical equation [6]; F=C/B; and F+M=Z=P/B [7]
	Q/B	2.514	Estimated by an empirical formula [3]
	EE	**0.811**	ME based on mass-balance principle
	Major preys	38,39,28,30,41	Based on the work of Roth [13]
**19**	**Spottail Shiner (*Notropis hudsonius*)**		
	B	**1.052**	Mass-balance approach with EE = 0.9 [1]
	P/B	0.940	Randall and Minns [14]
	Q/B	22.265	Estimated by an empirical formula [3]
	Major preys	46,40,41,48	Based on Hartman *et al.* [15]
**20**	**White sucker (*Catostomus commersonii*)**		
	B	0.014	SPM using time-series of catch and assessment gill net CPUE data of the lake [5]
	P/B	0.254	M estimated by an empirical equation [6]; F=C/B; and F+M=Z=P/B [7]
	Q/B	2.821	Estimated by an empirical formula [3]
	EE	**0.485**	ME based on mass-balance principle
	Major preys	41,46,49,40,42,47,39,43	Based on Becker [16]
**21**	**Black crappie (*Pomoxis nigromaculatus*)**		
	B	**0.077**	Mass-balance approach with EE = 0.9 [1]
	P/B	0.230	Randall and Minns [14]
	Q/B	2.346	Estimated by an empirical formula [3]
	Major preys	46,40,42,19,41,5,39	Adjusted based on Pearse [17]
**22**	**Burbot (*Lota lota*)**		
	B	0.679	SPM using time-series of catch and assessment gill net CPUE data of the lake [5]. The model also included Accumulated Degree Days (ADD) in the estimation.
	P/B	0.317	M estimated by an empirical equation [6]; F=C/B; and F+M=Z=P/B [7]
	Q/B	2.317	Estimated by an empirical formula [3]
	EE	**0.473**	ME based on mass-balance principle
	Major preys	5,38,6,30,28,19	From Persell and Ware [18]
**23**	**Trout perch (*Percopsis omiscomaycus*)**		
	B	**0.019**	Mass-balance approach with EE = 0.9 [1]
	P/B	0.690	Randall and Minns [14]
	Q/B	6.275	Estimated by an empirical formula [3]

	Major preys	46,40,39,42	Based on study of Nelson and Dick [19]
**24**	**Carp (*Cyprinus carpio*)**		
	B	**0.047**	Mass-balance approach with EE = 0.9 [1]
	P/B	0.110	Randall and Minns [14]
	Q/B	10.703	Estimated by an empirical formula [3]
	Major preys	41,40,39,47,42,44	Based on a couple of studies [16, 17]
**25**	**Largemouth bass (*Micropterus salmoides*)**		
	B	**0.090**	Mass-balance approach with EE = 0.8 [1]
	P/B	0.160	Randall and Minns [14]
	Q/B	2.386	Estimated by an empirical formula [3]
	Major preys	29,5,7,27,6,21,37	Excerpted from the study of Pearse [20],Pearse [21], and Snow [22]
**26**	**Bowfin (*Amia calva*)**		
	B	**0.013**	Mass-balance approach with EE = 0.6 [1]
	P/B	0.080	Randall and Minns [14]
	Q/B	1.971	Estimated by an empirical formula [3]
	Major preys	29,38,5,21,32,6	From the study of Lagler and Applegate [23]
**27**	**Bullhead (*Ameiurus nebulosus*)**		
	B	**0.103**	Mass-balance approach with EE = 0.9 [1]
	P/B	0.290	Randall and Minns [14]
	Q/B	2.244	Estimated by an empirical formula [3]
	Major preys	42,45,40,28,41,44	Excerpt from the studies of Pearse [21] and Kline and Wood [24]
**28**	**Others Shiners/minnows**[i]		
	B	**1.802**	Mass-balance approach with EE = 0.9 [1]
	P/B	1.006	Average of 5 species of Shiners, 4 species of Dace and 3 species of Minnows taken from Randall and Minns [14]
	Q/B	13.000	Average Q/B of mimic shiners, bluntnose minnows and pearl dace estimated by an empirical formula [3]
	Major preys	40,50,41,48,46	Based on analysis of Moyle [25]
**29**	**Sunfish (*Lepomis spp.*)**[ii]		
	B	**0.702**	Mass-balance approach with EE = 0.9 [1]
	P/B	0.577	Average of bluegill, pumpkinseed and green sunfish taken from the study of Randall and Minns [14]
	Q/B	3.710	Average Q/B of bluegill, pumpkinseed, and green sunfish estimated by an empirical formula [3]

	Major preys	41,44,39,42,46,40	Based on Etnier [26] and Phillips *et al.* [27]
**30**	**Darters (*Etheostoma spp.*)[iii]**		
	B	**1.381**	Mass-balance approach with EE = 0.9 [1]
	P/B	1.200	Randall and Minns [14]
	Q/B	10.720	Estimated by an empirical formula [3]
	Major preys	40,41,39,46	Based on Martin [28]
**31**	**Sculpins (*Cottus spp.*)[iv]**		
	B	**0.053**	Mass-balance approach with EE = 0.9 [1]
	P/B	0.770	Average of mottled and slimy sculpin from Randall and Minns [14]
	Q/B	5.910	Estimated by an empirical formula [3]
	Major preys	40,42,41,46	Based on Hershey [29] and Hondorp *et al.* [30]
**32**	**Other fishes[v]**		
	B	**1.621**	Mass-balance approach with EE = 0.9 [1]
	P/B	0.596	Average of P/B of seven species that comprised the group taken from Randall and Minns [14]
	Q/B	5.311	Q/B estimated for all the seven species using [3] and the average was taken
	Major preys	46,40,39,48,41	Obtained from a number of studies [31-33]
**33**	**Otter and Mink**		
	B	0.000	Guesstimate
	P/B	0.080	Guesstimate
	Q/B	41.975	Based on the data of Environment Protection Division, BC [34]
	EE	**0.000**	ME based on mass-balance principle
	Major preys	22,20,3,7,51,29,9,10,32	Based on Knudsen and Hale [35] and Erlinge [36]_ENREF_6
**34**	**Double-crested cormorant**		
	B	0.000	Assumed a negligible biomass as a place holder
	P/B	0.100	Database [37]
	Q/B	61.056	Database [37]
	EE	**0.152**	ME based on mass-balance principle
	Major preys	7,6,17,19,14,15,22,2	Based on the study of Hobson *et al.* [38] and Rudstam *et al.* [39]
**35**	**Other piscivorous birds**		
	B	0.000	Based on McDowell [40] and Patrick Schmalz, MNDNR, pers. comm.
	P/B	0.100	Assumed to be same as double-crested cormorant

	Q/B	74.000	Based on the data of Environment Protection Division, BC [34]
	EE	**0.198**	ME based on mass-balance principle
	Major preys	22,32,19,28,51,7,3,23	Mainly based on Manuel [41] and Kozie and Anderson [42]
**36**	**Turtle (*Chrysemys picta*)**		
	B	**0.000**	Mass-balance approach with EE = 0.8 [1]
	P/B	0.100	Guesstimate
	Q/B	3.500	Guesstimate
	Major preys	41,44,48,40,39	Based on Cooley *et al.* [43]_ENREF_4
**37**	**Frog**		
	B	**0.055**	Mass-balance approach with EE = 0.8 [1]
	P/B	3.139	Based on Deutschman and Peterka [44]
	**Q/B**	**10.463**	ME by providing P/Q=0.3
	Major preys	41,51,48,47,40	Based on Linzey [45]
**38**	**Crayfish**		
	B	**1.350**	Mass-balance approach with EE = 0.4 [1]
	P/B	5.500	Adopted from Momot and Gowing [46] and Momot [47]
	Q/B	36.000	From Ecopath model [48]
	Major preys	50	Based on Harris [49] and Madden *et al.* [50]
**39**	**Hex mayflies**		
	B	1.380	Based on Mille Lacs Lake survey
	P/B	6.400	Based on [18]
	Q/B	54.600	Adjusted based on study by Cummins and Klug [51] and Ross and Wallace [52]
	EE	**0.748**	ME based on mass-balance principle
	Major preys	50,49,48	Based on Koslucher and Minshall [53]
**40**	**Diptera**		
	B	**3.447**	Mass-balance approach with EE = 0.9 [1]
	P/B	9.000	Based on Breys's database [54]
	Q/B	54.600	Assumed to be same as other insects
	Major preys	50,49,46	Based on Koslucher and Minshall [53]
**41**	**Other insects**		
	B	**3.310**	Mass-balance approach with EE = 0.9 [1]
	P/B	4.225	Based on Breys's database [54]

	Q/B	54.600	Adjusted based on [52]
	Major preys	50,49,46,48	Adjusted from several studies [53, 55, 56]
**42**	**Amphipoda (*Gammarus spp.*)**		
	B	**0.291**	Mass-balance approach with EE = 0.9 [1]
	P/B	9.000	From Persell and Ware [18]
	Q/B	21.000	Based on Poepperl [57]
	Major preys	50,49	Based on the study of Koslucher and Minshall [53] and Marchant and Hynes [58]
**43**	**Pelecyopods**		
	B	**0.410**	Mass-balance approach with EE = 0.9 [1]
	P/B	4.000	From Persell and Ware [18]
	Q/B	8	From Brey [54] for bivalve of Ohio River
	Major preys	49,50,56	Adjusted from Nichols and Garling [59]
**44**	**Gastropods**		
	B	**0.513**	Mass-balance approach with EE = 0.9 [1]
	P/B	4.000	From Persell and Ware [18]
	Q/B	14.554	Based on Brey's database [54]
	Major preys	48,50,47	Loosely based on Madsen [60]
**45**	**Oligochaete (*Hirudinea*)**		
	B	**0.152**	Mass-balance approach with EE = 0.9 [1]
	P/B	3.000	From Persell and Ware [18]
	Q/B	40.000	Based on Poepperl [57]
	Major preys	49,40,44,42,45	Based on Bradley and Reynolds [61]
**46**	**Zooplankton**		
	B	7.700	Estimated from the Lake survey data
	P/B	12.000	From the range suggested by [62]
	Q/B	124.228	Based on Gulati *et al.* [63]
	EE	**0.830**	ME based on mass-balance principle
	Major preys	49	Considered solely herbivorous
**47**	**Macrophytes**		
	B	**0.588**	Mass-balance approach with EE = 0.5 [1]
	P/B	1.780	Based on Rich *et al.* [64]
**48**	**Periphyton**		
	B	**1.906**	Mass-balance approach with EE = 0.5 [1]
	P/B	40.000	From Angelini *et al.* [65]

**49**	**Phytoplankton**		
	B	38.000	Approximated based on Chlorophyll a data
	P/B	113.000	Mean P/B from fresh water ecosystem [62]
**50**	**Detritus**		
	B	300.000	From Ontario food web model Halfon and Schito [66]

Diet matrix of Ecopath with Ecosim model

SrN	Prey/Predators	1	2	3	4	5	6	7	8	9	10	11	12
1	Walleye age 0			0.000	0.000				0.020	0.005	0.001	0.020	0.008
2	Walleye age 1			0.003	0.001					0.095	0.050		0.020
3	Walleye age 2				0.004						0.030		0.020
4	Walleye age 3+				0.004						0.029		0.008
5	Yellow perch age 0	0.650	0.441	0.345	0.242		0.070	0.364	0.320	0.188	0.025	0.312	0.005
6	Yellow perch age 1		0.098	0.148	0.108			0.029	0.011	0.150	0.020	0.011	0.011
7	Yellow perch age 2+				0.010					0.225	0.325		0.231
8	Northern pike age 0										0.000		0.009
9	Northern pike age 1				0.000								0.027
10	Northern pike age 2+												0.009
11	Muskellunge age 0								0.000	0.000			
12	Muskellunge age 1+												
13	Cisco age 0		0.015	0.015	0.003			0.007	0.038	0.005	0.005	0.038	0.004
14	Cisco age 1				0.011					0.020	0.040		0.054
15	Cisco age 2+				0.010					0.025	0.049		0.119
16	Smallmouth bass age 0	0.000			0.000			0.000	0.001	0.000	0.000	0.005	
17	Smallmouth bass age 1+										0.001		0.080
18	Rock bass	0.001	0.001	0.000	0.000				0.032	0.010	0.009	0.017	0.034
19	Spottail shiner	0.100	0.027	0.099	0.080		0.001	0.001	0.020	0.050	0.099	0.001	0.001
20	White sucker	0.000	0.000	0.000					0.001	0.001	0.001	0.079	0.161
21	Black crappie								0.001	0.050	0.010	0.010	0.010
22	Burbot				0.013						0.001		0.001
23	Trout-perch		0.020		0.000				0.001	0.001	0.001	0.001	0.001
24	Common carp								0.035			0.028	0.056
25	Largemouth bass				0.002				0.010			0.010	0.020
26	Bowfin										0.001		
27	Bullhead												0.001
28	O. Shiners/minnows	0.100	0.246	0.171	0.187		0.001	0.001	0.037	0.038	0.048	0.037	
29	Sunfish	0.034	0.022	0.017	0.050				0.015	0.005	0.005	0.015	0.030
30	Darters	0.070	0.020	0.002	0.004		0.097	0.187	0.070	0.038	0.034	0.056	0.040
31	Sculpins			0.001	0.005								
32	Other fishes		0.098	0.044	0.080				0.008	0.075	0.206	0.008	
33	Otter and Mink												
34	D.C. cormorants												
35	O. piscivorous birds												
36	Turtles												0.017
37	Frogs				0.021				0.020	0.010	0.010	0.020	0.011
38	Crayfish	0.000	0.001	0.015	0.048		0.243	0.260	0.066	0.010		0.065	0.013
39	Hex mayflies	0.005	0.004	0.132	0.105		0.250	0.019	0.075			0.074	
40	Diptera	0.002	0.002	0.002	0.005	0.045	0.054	0.019	0.025			0.025	
41	Other insects	0.000	0.000	0.001	0.001	0.001	0.071	0.004	0.025			0.025	
42	Amphipoda	0.000	0.000	0.004	0.004	0.013	0.000	0.001					
43	Pelecyopoda			0.000			0.186	0.102	0.010			0.010	
44	Gastropoda	0.000	0.003	0.000	0.001		0.009	0.005	0.010			0.010	
45	Oligochaete	0.001	0.001	0.000	0.001				0.040			0.040	
46	Zooplankton	0.036				0.941	0.018	0.001	0.108			0.084	
47	Macrophytes												
48	Periphyton												
49	Phytoplankton												
50	Detritus												
51	Import												

SrN	Prey/Predators	13	14	15	16	17	18	19	20	21	22	23	24
1	Walleye age 0												
2	Walleye age 1												
3	Walleye age 2												
4	Walleye age 3+												
5	Yellow perch age 0		0.020	0.020		0.020	0.020			0.080	0.299	0.012	
6	Yellow perch age 1					0.020	0.020				0.155		
7	Yellow perch age 2+												
8	Northern pike age 0												
9	Northern pike age 1												
10	Northern pike age 2+												
11	Muskellunge age 0												
12	Muskellunge age 1+												
13	Cisco age 0												
14	Cisco age 1												
15	Cisco age 2+												
16	Smallmouth bass age 0												
17	Smallmouth bass age 1+												
18	Rock bass												
19	Spottail shiner				0.001	0.001	0.001			0.090	0.060		
20	White sucker										0.000		
21	Black crappie				0.165								
22	Burbot					0.010							
23	Trout-perch										0.000		
24	Common carp												
25	Largemouth bass				0.001								
26	Bowfin												
27	Bullhead												
28	O. Shiners/minnows				0.001	0.003	0.025			0.001	0.071		
29	Sunfish				0.080	0.090	0.010						
30	Darters				0.515	0.002	0.025				0.120		
31	Sculpins										0.005		
32	Other fishes				0.002	0.003					0.040		
33	Otter and Mink												
34	D.C. cormorants												
35	O. piscivorous birds												
36	Turtles												
37	Frogs												
38	Crayfish		0.015	0.030	0.002	0.802	0.788				0.219	0.008	0.001
39	Hex mayflies		0.147	0.158	0.018	0.003	0.087		0.086	0.060		0.082	0.150
40	Diptera	0.200	0.172	0.158	0.072		0.000	0.220	0.133	0.200	0.010	0.247	0.150
41	Other insects		0.147	0.158	0.066	0.033	0.024	0.031	0.171	0.080	0.010		0.349
42	Amphipoda				0.043				0.121	0.110	0.011	0.069	0.069
43	Pelecyopoda		0.052	0.158					0.063				
44	Gastropoda		0.041	0.158					0.040				0.069
45	Oligochaete				0.002	0.013							0.050
46	Zooplankton	0.800	0.407	0.158	0.032		0.000	0.737	0.142	0.380		0.582	0.050
47	Macrophytes								0.102				0.113
48	Periphyton							0.012					
49	Phytoplankton								0.142				
50	Detritus												
51	Import												

SrN	Prey/Predators	25	26	27	28	29	30	31	32	33	34	35	36
1	Walleye age 0	0.001									0.010	0.003	
2	Walleye age 1	0.001									0.030	0.008	
3	Walleye age 2									0.100	0.010	0.040	
4	Walleye age 3+									0.045	0.010	0.016	
5	Yellow perch age 0	0.163	0.099								0.001	0.001	0.001
6	Yellow perch age 1	0.102	0.045								0.253	0.027	0.001
7	Yellow perch age 2+	0.143	0.001							0.066	0.312	0.094	0.002
8	Northern pike age 0											0.001	
9	Northern pike age 1									0.060	0.007	0.001	
10	Northern pike age 2+									0.060	0.007	0.010	
11	Muskellunge age 0									0.000	0.000		
12	Muskellunge age 1+									0.010			
13	Cisco age 0										0.020		
14	Cisco age 1										0.061		
15	Cisco age 2+									0.030	0.060		
16	Smallmouth bass age 0												
17	Smallmouth bass age 1+									0.002	0.099		
18	Rock bass											0.033	
19	Spottail shiner	0.010		0.011							0.061	0.134	
20	White sucker									0.120		0.010	
21	Black crappie	0.060	0.080								0.020		0.001
22	Burbot									0.223	0.035	0.144	
23	Trout-perch										0.001	0.040	
24	Common carp		0.001									0.010	
25	Largemouth bass		0.001										
26	Bowfin	0.001	0.001										
27	Bullhead	0.120	0.032							0.021			0.001
28	O. Shiners/minnows	0.027	0.001	0.097						0.043		0.130	
29	Sunfish	0.168	0.464							0.060		0.001	0.001
30	Darters										0.000		
31	Sculpins												
32	Other fishes	0.015	0.077							0.052	0.001	0.144	
33	Otter and Mink												
34	D.C. cormorants									0.000			
35	O. piscivorous birds									0.002			0.001
36	Turtles												
37	Frogs	0.057	0.014							0.012			0.001
38	Crayfish	0.003	0.182	0.010		0.010				0.001	0.000	0.005	0.001
39	Hex mayflies	0.001		0.012	0.010	0.100	0.190	0.005	0.075				0.023
40	Diptera	0.046		0.216	0.268	0.041	0.500	0.760	0.378				0.023
41	Other insects	0.036	0.001	0.065	0.166	0.484	0.220	0.050	0.049	0.032		0.015	0.365
42	Amphipoda	0.026		0.326	0.033	0.051	0.029	0.120				0.010	
43	Pelecyopoda			0.001		0.010	0.001	0.010		0.001			
44	Gastropoda			0.036		0.254	0.001	0.010					0.296
45	Oligochaete	0.001	0.001	0.226			0.001	0.010					
46	Zooplankton	0.020		0.001	0.123	0.050	0.059	0.035	0.438				
47	Macrophytes												
48	Periphyton				0.143				0.056				0.283
49	Phytoplankton												
50	Detritus				0.258								
51	Import								0.004	0.060		0.125	

SrN	Prey/Predators	37	38	39	40	41	42	43	44	45	46
1	Walleye age 0										
2	Walleye age 1										
3	Walleye age 2										
4	Walleye age 3+										
5	Yellow perch age 0										
6	Yellow perch age 1										
7	Yellow perch age 2+										
8	Northern pike age 0										
9	Northern pike age 1										
10	Northern pike age 2+										
11	Muskellunge age 0										
12	Muskellunge age 1+										
13	Cisco age 0										
14	Cisco age 1										
15	Cisco age 2+										
16	Smallmouth bass age 0										
17	Smallmouth bass age 1+										
18	Rock bass										
19	Spottail shiner					0.001					
20	White sucker										
21	Black crappie										
22	Burbot										
23	Trout-perch										
24	Common carp										
25	Largemouth bass										
26	Bowfin										
27	Bullhead										
28	O. Shiners/minnows					0.001					
29	Sunfish										
30	Darters										
31	Sculpins										
32	Other fishes					0.001					
33	Otter and Mink										
34	D.C. cormorants										
35	O. piscivorous birds										
36	Turtles										
37	Frogs										
38	Crayfish										
39	Hex mayflies	0.002	0.010								
40	Diptera	0.035	0.007			0.010				0.291	
41	Other insects	0.602	0.001			0.010					
42	Amphipoda									0.100	
43	Pelecyopoda										
44	Gastropoda	0.018								0.140	
45	Oligochaete	0.010								0.050	
46	Zooplankton				0.100	0.100		0.128			
47	Macrophytes	0.070	0.001						0.050		
48	Periphyton	0.080	0.001	0.136		0.098			0.800		
49	Phytoplankton			0.341	0.410	0.291	0.350	0.614		0.419	1.000
50	Detritus		0.980	0.523	0.490	0.489	0.650	0.258	0.150		
51	Import	0.183									

References

1. Christensen V, Walters CJ. Ecopath with Ecosim: methods, capabilities and limitations. Ecol Model. 2004;172(2-4):109-39.

2. Drake MT. Mille Lacs Lake Walleye Safe Harvest Estimation for the 2007 Fishing Season. Minnesota Department of Natural Resources (MNDNR), Minnesota; 2007. Contract No.: Report.

3. Palomares MLD, Pauly D. Predicting food consumption of fish populations as functions of mortality, food type, morphometrics, temperature and salinity. Marine and freshwater research. 1998;49(5):447-53.

4. Tarby MJ. Characteristics of yellow perch cannibalism in Oneida Lake and the relation to first year survival. Trans Am Fish Soc. 1974;103(3):462-71.

5. Hilborn R, Mangel M. The ecological detective: confronting models with data: Princeton Univ. Press; 1997. 330 p.

6. Pauly D. On the interrelationships between natural mortality, growth parameters, and mean environmental temperature in 175 fish stocks. ICES J Mar Sci. 1980;39(2):175.

7. Allen KR. Relation between production and biomass. Journal of the Fisheries Research Board of Canada. 1971;28(10):1573-81.

8. Hourston AS. The food and growth of the maskinonge (Esox masquinongy Mitchill) in Canadian waters. Journal of the Fisheries Research Board of Canada. 1952;8:347-68.

9. Engel S. Food habits and prey selection of coho salmon (Oncorhynchus kisutch) and cisco (Coregonus artedii) in relation to zooplankton dynamics in Pallette Lake, Wisconsin. Trans Am Fish Soc. 1976;105(5):607-14.

10. Kumar R, Martell SJ, Pitcher TJ, Varkey DA. Temperature-Driven Decline of a Cisco Population in Mille Lacs Lake, Minnesota. North Am J Fish Manage. 2013;33(4):669-81.

11. Pelham ME, Pierce CL, Larscheid JG. Diet dynamics of the juvenile piscivorous fish community in Spirit Lake, Iowa, USA, 1997-1998. Ecol Freshw Fish. 2001;10(4):198-211.

12. Frey AP, Bozek MA, Edwards CJ, Newman SP. Diet overlap and predation between smallmouth bass and walleye in a north temperate lake. J Freshw Ecol. 2003;18(1):43-54.

13. Roth BM. The role of competition, predation, and their interaction in invasion dynamics: predator accelerated replacement: University of Wisconsin, Madison; 2001.

14. Randall R, Minns C. Use of fish production per unit biomass ratios for measuring the productive capacity of fish habitats. Can J Fish Aquat Sci. 2000;57(8):1657-67.

15. Hartman KJ, Vondracek B, Parrish DL, Muth KM. Diets of emerald and spottail shiners and potential interactions with other western Lake Erie planktivorous fishes. J Gt Lakes Res. 1992;18(1):43-50.

16. Becker GC. Fishes of Wisconsin: The University of Wisconsin Press, Madison; 1983.

17. Pearse AS. The food of the shore fishes of certain Wisconsin lakes. Bulletin of the United States Bureau of Fisheries. 1918;37:249-92.

18. Persell JS, Ware TM. A Study of the Productivity of Mille Lacs Lake 1981. Minnesota: Mille Lacs Reservation Department of Conservation; 1982. Contract No.: Report.

19. Nelson PA, Dick TA. Factors shaping the parasite communities of trout-perch, Percopsis omiscomaycus Walbaum (Osteichthyes: Percopsidae), and the importance of scale. Canadian journal of zoology. 2002;80(11):1986-99.

20. Pearse AS. Distribution and food of the fishes of Green lake, Wisconsin, in summer. Bulletin of the United States Bureau of Fisheries. 1922;37:253-72.

21. Pearse AS. Amount of food eaten by four species of fresh-waters fishes. Ecology. 1924;5(3):254-8.

22. Snow HE. Harvest and feeding habits of largemouth bass in Murphy Flowage, Wisconsin. Department of Natural Resources, Madison, Wisconsin; 1971.

23. Lagler KF, Applegate VC. Further studies of the food of the bowfin (Amia calva) in southern Michigan, with notes on the inadvisability of using trapped fish in food analyses. Copeia. 1942;1942(3):190-1.

24. Kline JL, Wood BM. Food habits and diet selectivity of the brown bullhead. Journal of freshwater ecologyLa Crosse, WI. 1996;11(2):145-51.

25. Moyle PB. Ecological segregation among three species of minnows (Cyprinidae) in a Minnesota lake. Trans Am Fish Soc. 1973;102(4):794-805.

26. Etnier DA. Food of three species of sunfishes (Lepomis, Centrarchidae) and their hybrids in three Minnesota lakes. Trans Am Fish Soc. 1971;100(1):124-8.

27. Phillips GL, Schmid WD, Underhill JC. Fishes of the Minnesota region: Univ of Minnesota Press, Minneapolis, Minnesota; 1982. 248 p.

28. Martin FD. Diets of four sympatric species of Etheostoma (Pisces: Percidae) from southern Indiana: interspecific and intraspecific multiple comparisons. Environmental Biology of Fishes. 1984;11(2):113-20.

29. Hershey AE. Effects of predatory sculpin on the chironomid communities in an arctic lake. Ecology. 1985;66(4):1131-8.

30. Hondorp DW, Pothoven SA, Brandt SB. Influence of Diporeia density on diet composition, relative abundance, and energy density of planktivorous fishes in southeast Lake Michigan. Trans Am Fish Soc. 2005;134(3):588-601.

31. Keast A. Development of dietary specializations in a summer community of juvenile fishes. Environmental Biology of Fishes. 1985;13(3):211-24.

32. Markley MH. Notes on the Food Habits and Parasites of the Stickleback, Gasterosteus aculeatus (Linnaeus), in the Sacramento River, California. Copeia. 1940;1940(4):223-5.

33. Johnson JH, Dropkin DS. Diel variation in diet composition of a riverine fish community. Hydrobiologia. 1993;271(3):149-58.

34. Warrington PD. Animal weights and their food and water requirements BC, Canada: Environment and Resource Division, Ministry of Environment, Government of British Columbia; 1996 [updated September 17, 2001. Available from: http://www.env.gov.bc.ca/wat/wq/reference/foodandwater.html.

35. Knudsen GJ, Hale JB. Food habits of otters in the Great Lakes region. The Journal of Wildlife Management. 1968;32:89-93.

36. Erlinge S. Food habits of the otter Lutra lutra L. and the mink Mustela vison Schreber in a trout water in southern Sweden. Oikos. 1969:1-7.

37. Nelson B. Seabirds, Their Biology and Ecology. New York: A&S Publishers Inc.; 1979. 219 p.

38. Hobson KA, Knapton RW, Lysack W. Population, diet and reproductive success of Double-crested Cormorants breeding on Lake Winnipegosis, Manitoba, in 1987. Colonial Waterbirds. 1989;12(2):191-7.

39. Rudstam LG, VanDeValk AJ, Adams CM, Coleman JTH, Forney JL, Richmond ME. Cormorant predation and the population dynamics of walleye and yellow perch in Oneida Lake. Ecol Appl. 2004;14(1):149-63.

40. McDowell M. Mille Lacs National Wildlife Refuge- 2007 Report. Minnesota: Minnesota Department of Natural Resources; 2007. Contract No.: Report.

41. Manuel CG. The relation of gulls and terns to the commercial fisheries of Saginaw Bay, Michigan, with particular reference to the Common Tern, Sterna hirundo Linnaeus: Univ. of Michigan, Ann Arbor; 1931.

42. Kozie KD, Anderson RK. Productivity, diet, and environmental contaminants in bald eagles nesting near the Wisconsin shoreline of Lake Superior. Archives of Environmental Contamination and Toxicology. 1991;20(1):41-8.

43. Cooley CR, Floyd AO, Dolinger A, Tucker PB. Demography and diet of the painted turtle (Chrysemys picta) at high-elevation sites in southwestern Colorado. The Southwestern Naturalist. 2003:47-53.

44. Deutschman MR, Peterka JJ. Secondary production of tiger salamanders (Ambystoma tigrinum) in three North Dakota prairie lakes. Can J Fish Aquat Sci. 1988;45(4):691-7.

45. Linzey DW. Food of the leopard frog, Rana p. pipiens, in central New York. Herpetologica. 1967;23(1):11-7.

46. Momot W, Gowing H, editors. The cohort production and life cycle turnover ratio of the crayfish, Orconectes virilis, in three Michigan lakes. Freshwater Crayfish, 2nd International Crayfish Symposium Louisiana State University, Baton Rouge; 1975.

47. Momot WT. Crayfish production: a reflection of community energetics. Journal of Crustacean Biology. 1984;4(1):35-54.

48. Mavuti K, Moreau J, Munyandorero J, Plisnier PD. Analysis of trophic relationships in two shallow equatorial lakes Lake Naivasha (Kenya) and Lake Ihema (Rwanda) using a multispecifies trophic model. Hydrobiologia. 1996;321(2):89-100.

49. Harris LD. The nature of cumulative impacts on biotic diversity of wetland vertebrates. Environ Manage. 1988;12(5):675-93.

50. Madden JD, Finerty MW, Grodner RM. Survey of persistent pesticide residues in the edible tissues of wild and pond-raised Louisiana crayfish and their habitat. Bulletin of environmental contamination and toxicology. 1989;43(5):779-84.

51. Cummins KW, Klug MJ. Feeding ecology of stream invertebrates. Annual Review of Ecology and Systematics. 1979;10(1):147-72.

52. Ross DH, Wallace JB. Longitudinal patterns of production, food consumption, and seston utilization by net-spinning caddisflies (Trichoptera) in a southern Appalachian stream (USA). Holarctic Ecology. 1983;6(3):270-84.

53. Koslucher DG, Minshall GW. Food habits of some benthic invertebrates in a northern cool-desert stream (Deep Creek, Curlew Valley, Idaho-Utah). Transactions of the American Microscopical Society. 1973;92(3):441-52.

54. Brey T. Population dynamics in benthic invertebrates. A virtual handbook. http://www.thomas-brey.de/science/virtualhandbook2001. Available from: http://www.thomas-brey.de/science/virtualhandbook.

55. Mecom JO. Feeding habits of Trichoptera in a mountain stream. Oikos. 1972;23(3):401-7.

56. Monson M, Holzenthal R, Flint Jr O, editors. The caddisflies of the Lake Itasca region of Minnesota (Insecta: Trichoptera). Proceedings of the 8th International Symposium on Trichoptera; 1995.

57. Poepperl R. A quantitative food web model for the macroinvertebrate community of a northern German lowland stream. Int Rev Hydrobiol. 2003;88(5):433-52.

58. Marchant R, Hynes HBN. Field estimates of feeding rate for Gammarus pseudolimnaeus (Crustacea: Amphipoda) in the Credit River, Ontario. Freshwater Biol. 1981;11:147-56.

59. Nichols SJ, Garling D. Food-web dynamics and trophic-level interactions in a multispecies community of freshwater unionids. Canadian journal of zoology. 2000;78(5):871-82.

60. Madsen H. Food selection by freshwater snails in the Gezira irrigation canals, Sudan. Hydrobiologia. 1992;228(3):203-17.

61. Bradley MDK, Reynolds JD. Diet of the leeches Erpobdella octoculata(L) and Helobdella stagnalis(L) in a lotic habitat subject to organic pollution. Freshw Biol. 1987;18(2):267-75.

62. Wetzel R. Limnology, 3 E. Lake and River Ecosystems. Academic Press, 525 B Street, Ste1900, San Diego, CA 92101, USA850. 2001:850.

63. Gulati RD, Siewertsen K, Postema G. The zooplankton: its community structure, food and feeding, and role in the ecosystem of Lake Vechten. Hydrobiologia. 1982;95(1):127-63.

64. Rich PH, Wetzel RG, Thuy NV. Distribution, production and role of aquatic macrophytes in a southern Michigan marl lake. Freshw Biol. 1971;1(1):3-21.

65. Angelini R, Agostinho AA, Gomes LC. Modeling energy flow in a large Neotropical reservoir: a tool do evaluate fishing and stability. Neotropical Ichthyology. 2006;4:253-60.

66. Halfon E, Schito N, editors. Trophic Models of Aquatic Ecosystems. ICLARM Conference Proceedings; 1993; ICLARM, Manila.

---

[i] This group includes Mimic shiner (*Notropis volucellus*), Golden shiner (*Notemigonus crysoleucas*), Blacknose shiner (*Notropis heterolepis*), Blacknose dace (*Rhinichthys atratulus*), Pearl dace (*Semotilus margarita*), Northern redbelly dace (*Phoxinus eos*), Finescale dace (*Phoxinus neogaeus*), Bluntnose minnow (*Pimephales notatus*), Brassy minnow (*Hybognathus hankinsoni*), Fathead minnow (*Pimephales promelas*), Central mudminnow (*Umbra lima*)

[ii] This group includes Bluegill (*Lepomis macrochirus*), Pumpkinseed (*Lepomis gibbosus*), Green sunfish (*Lepomis cyanellus*)

[iii] This group includes Bluegill (*Lepomis macrochirus*,) Pumpkinseed (*Lepomis gibbosus*), Green sunfish (*Lepomis cyanellus*)

[iv] This group has Mottled sculpin (*Cottus bairdi*), Slimy sculpin (*Cottus cognatus*)

[v] This group includes all those fish species in the lake which were not included in any other functional group in the model such as Banded killifish (*Fundulus diaphanus*), Tadpole madtom (*Noturus gyrinus*), Shorthead redhorse (*Moxostoma macrolepidotum*), Brook stickleback (*Culaea inconstans*), Brook silverside (*Labidesthes sicculus*), Silver redhorse (*Moxostoma spp*.), Logperch (*Percina caprodes*).
