# Supplementary figures and images for: Ecosystem approach to fisheries: Exploring environmental and trophic effects on Maximum Sustainable Yield (MSY) reference point estimates
Source: PLoS One. 2017 Sep 28;12(9):e0185575. doi: 10.1371/journal.pone.0185575 (PMC5619794; doi:10.1371/journal.pone.0185575)

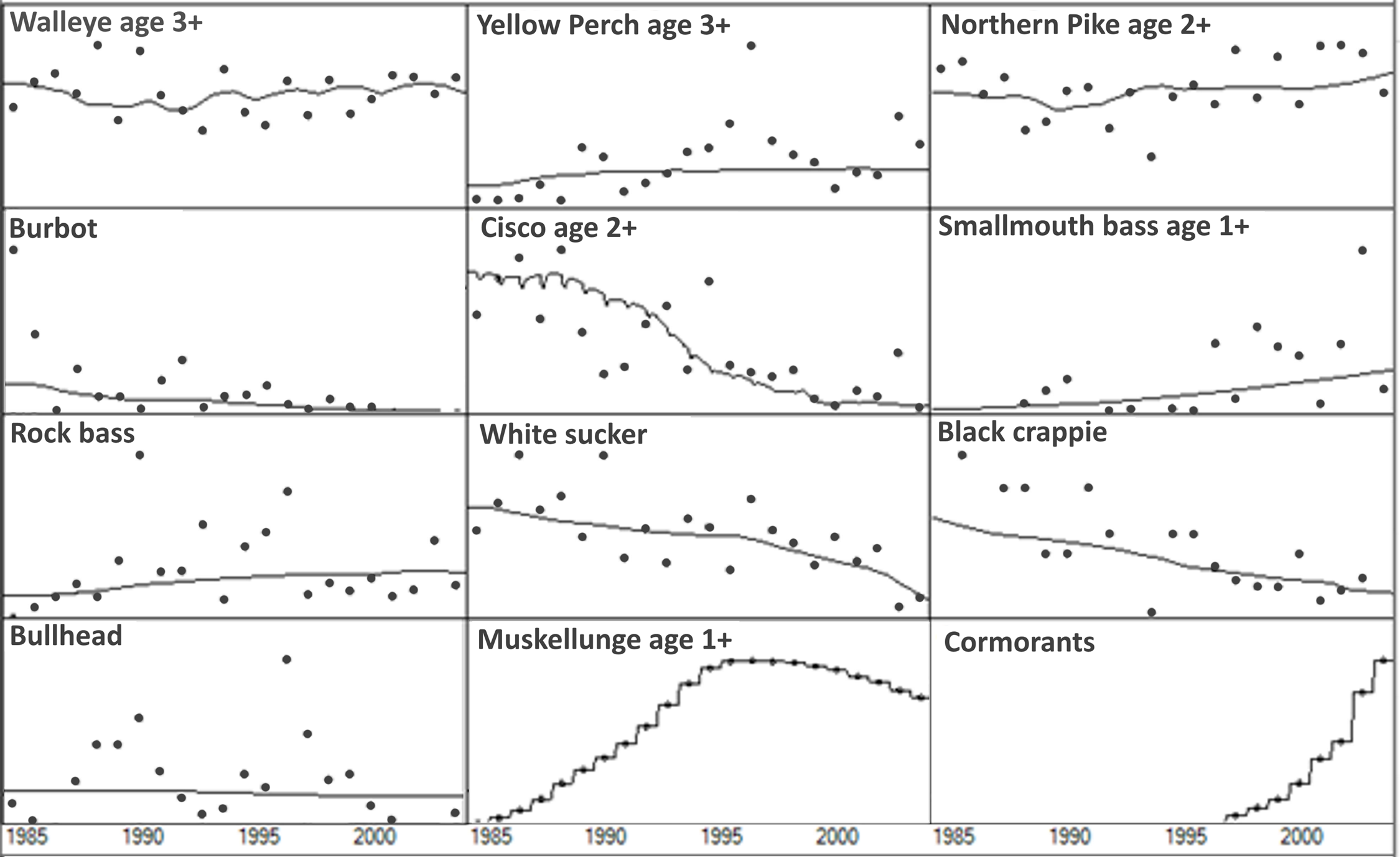

Supplement: S1 Fig — The dots are the observations and the lines are the Ecosim predictions. Prediction lines that exactly follow the dots are forced biomasses. (TIF) [file pone.0185575.s002.tif]
